# Herbal medicine approach to relieving dyspnea: A narrative review of efficacy and mechanisms

**DOI:** 10.22038/ijbms.2025.85518.18486

**Published:** 2025

**Authors:** Mahboobeh Ghasemzadeh Rahbardar, Mohammad Hossein Boskabady

**Affiliations:** 1 Clinical Research Development Unit, Shahid Hasheminejad Hospital, Mashhad University of Medical Sciences, Mashhad, Iran; 2 Applied biomedical Research Center, Basic Sciences Research Institute, Mashhad University of Medical Sciences, Mashhad, Iran; 3 Department of Physiology, Faculty of Medicine, Mashhad University of Medical Sciences, Mashhad, Iran

**Keywords:** Anti-Inflammatory agents, Anti-oxidants, Crocus, Curcuma, Garlic, Nigella sativa, Respiratory distress – syndrome, Rosmarinus

## Abstract

Dyspnea, a distressing symptom characterized by difficult or labored breathing, can be caused by a variety of underlying processes, including respiratory and cardiovascular problems. Despite advancements in medicine, a need remains for more effective dyspnea therapies. Herbal therapy has emerged as a viable approach in this field, with potential therapeutic benefits. The purpose of this narrative review is to assess the efficacy of herbal medication in reducing dyspnea. A comprehensive search was undertaken without time constraints utilizing Google Scholar, PubMed, and Scopus databases up to December 2024 to collect relevant clinical trials. Herbal medicine (*Allium sativum* L., *Carum copticum *(L.) Benth. & Hook.f., *Crocus sativus *L., *Curcuma longa* L., *Eucalyptus globulus* Labill., *Mentha* × *piperita* L., *Nigella sativa* L., *Rosmarinus officinalis* L., *Thymus vulgaris* L., and *Zataria multiflora* Boiss.) and their main components have been shown to reduce dyspnea through multiple mechanisms of disease, including anti-inflammatory, bronchodilatory, and anti-oxidant properties. The findings indicate that herbal remedies may be a useful complement or alternative therapy for managing dyspnea. It could be concluded that herbal therapy offers an effective approach to managing dyspnea, providing a natural and potentially beneficial option for people experiencing respiratory distress. More research and clinical trials are needed to understand the exact mechanisms of action and maximize the use of herbal therapies in the treatment of dyspnea.

## Introduction

**Figure 1:**
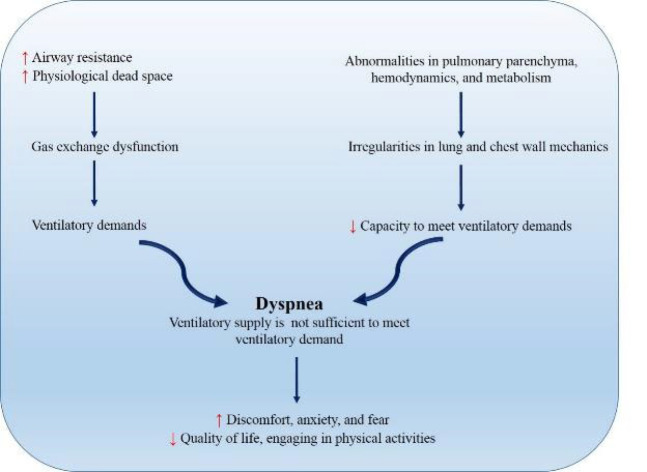
Pathophysiology and effect of dyspnea on individual’s life

Dyspnea, commonly known as shortness of breath, is a distressing symptom that can significantly impact a person’s quality of life. It may show across a spectrum of disorders, from heart failure and pulmonary embolism to respiratory disorders like asthma and chronic obstructive pulmonary disease (COPD) (1, 2). Dyspnea can induce discomfort, anxiety, and fear, limiting an individual’s capacity to engage in physical activities and consequently impacting their overall well-being (3, 4) (Figure 1).

The underlying mechanisms of dyspnea are complicated and extensive. It can originate from a mismatch between the body’s demand for oxygen and its ability to provide it, producing a cascade of physiological responses that involve the respiratory and cardiovascular systems. Impaired gas exchange, increased respiratory drive, and altered lung mechanics all contribute to the experience of breathlessness (5).

Methods used to assess dyspnea in humans include pulmonary function tests (PFTs), which evaluate lung function parameters such as forced expiratory volume in one second (FEV_1_) and forced vital capacity (FVC). These tests provide useful information about the respiratory capabilities of individuals and their relation to dyspnea (6, 7). Furthermore, exercise testing and arterial blood gas analysis are used to assess and define the severity of dyspnea and its influence on daily activities (8). Understanding the relationship between these physiological parameters and dyspnea perception enables a better understanding of the symptoms and suggests treatment strategies tailored to each individual.

Current dyspnea treatments primarily focus on addressing the underlying causes, alleviating symptoms, and restoring respiratory function. However, there remains an urgent need to develop new and more effective treatment options for this common condition.

In recent decades, researchers have increasingly focused on herbal medicine for the treatment of various diseases (9-12), including dyspnea (13) and other respiratory disorders (14-16). Additionally, according to the World Health Organization (WHO), 11% of medications come from plants, and 80% of people worldwide use phytochemistry to meet their basic health-related needs (17). 

Traditional folk medicine has historically used a wide range of herbs and natural remedies to treat respiratory and breathing problems (18-20). Avicenna’s “Canon of Medicine” (Al-Qanun fi al-Tibb) mentions various plants that have beneficial effects on respiratory disorders, including dyspnea. Avicenna classified plants according to their medical characteristics and frequently prescribed them for a variety of diseases, including respiratory disorders. Avicenna believed that several plants, including saffron (*Crocus sativus *L.) and garlic (*Allium sativum L.*), had significant advantages on the respiratory system (21). Similarly, Mohammad Hossein Aghili Khorasani’s “Makhzan al-Adwiya” (22) and “Tohfat al-Momenin” (23) contributed to the discourse of herbal medicine by describing a variety of botanical medicines and their applications in Persian medications. Indian medicine has long utilized herbs like turmeric (*Curcuma longa *L.) for their expectorant properties to alleviate respiratory congestion (24). These diverse herbal remedies from ancient medical systems reflect a profound understanding of the therapeutic properties of plants in managing respiratory disorders, underscoring the universal reliance on botanical medicine for respiratory health across various cultures and civilizations.

Pharmacological studies also illustrated that plants such as *Allium cepa* L. (25), *C. sativus* L. (26), *Ocimum basilicum* L. (27), *Portulaca oleracea* L. (28), and *Zataria multiflora* Boiss. (29) have long been used for their supposed respiratory advantages, which include anti-inflammatory, bronchodilatory, and expectorant properties.

The primary objective of this narrative review is to assess the efficacy of herbal medicine in treating dyspnea and to elucidate the various mechanisms by which these herbal therapies operate. By evaluating the therapeutic properties of herbal treatments, this review aims to identify alternative and complementary methods for managing dyspnea, potentially offering safer and more effective solutions for individuals with respiratory issues. The present review not only aims to improve our understanding of the potential of herbal treatments but also to pave the way for a broader approach to alleviating breathing problems, attracting researchers to design and formulate strategies that improve the safe integration of herbal remedies into medicine, bridging the gap between laboratory research and clinical use.

## Methods

To gather relevant information, an organized search was undertaken on Google Scholar, PubMed, and Scopus without time limits until December 2024. The search terms used were “herbal remedies,” “breathing difficulties,” “dyspnea,” “shortness of breath,” “breathlessness,” “respiratory distress,” “forced vital capacity,” “FVC,” “forced expiratory volume in one second,” “FEV_1_,” “pulmonary function tests,” “PFTs,” “herbal medicine,” and “natural remedies.”

**Figure 2:**
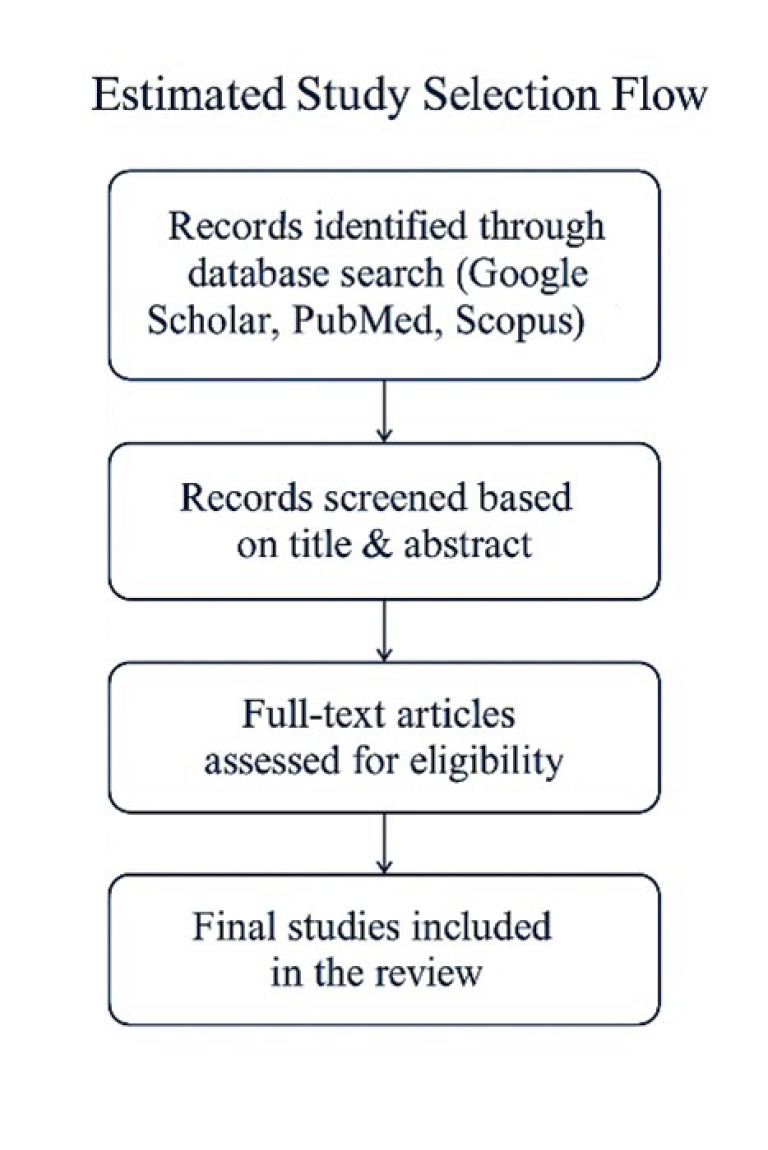
Study selection process for the narrative review on herbal remedies for dyspnea

The selection of relevant articles was conducted manually by a single reviewer, ensuring consistency in identifying studies related to herbal interventions for dyspnea. No automation tools were used in the screening or extraction process. The inclusion criteria covered peer-reviewed papers investigating the mechanisms and efficacy of herbal therapies in respiratory conditions, with a focus on English-language publications (at least the abstract). Studies unrelated to herbal treatments for dyspnea, non-peer-reviewed research, and articles falling outside this scope were excluded. The selection process followed a structured approach to ensure relevance and quality (Figure 2).

## Risk of bias consideration

Since this is a narrative review, a formal risk-of-bias assessment was not conducted. However, the quality of studies was evaluated based on peer-review status, relevance to herbal therapy for dyspnea, and study design considerations to ensure meaningful synthesis of findings.

### Herbal interventions for dyspnea

The effects of various medicinal plants, their derivatives, and other natural products on dyspnea were investigated, and most of these substances showed promising results. The effects of most natural products on dyspnea and the possible mechanism(s) are provided in this review. 

## Allium sativum L. (Garlic)

The Amaryllidaceae family includes garlic, formally known as *Allium sativum *L. (*A. sativum*), which is native to Asia but is now widely cultivated in regions such as China, Egypt, Europe, and Mexico. This plant is widely consumed in Iran, where traditional medicine uses its leaves, flowers, and bulbs. *A. sativum* is used externally to treat eczema, scabies, premature graying of the hair, inflammation associated with tetanus, and lung inflammation. It is also used to cure fever and coughing (30). Modern research has also revealed its advantageous properties, including anti-oxidant, immunomodulatory (31), anti-inflammatory (32), antimicrobial (33), and antitumor (34). The following section will explore the influence of garlic on dyspnea.

### The effects of A. sativum powder

The effectiveness of garlic powder in improving arterial oxygenation and dyspnea was examined in a study that involved subjects with hepatopulmonary syndrome. Patients self-administered garlic powder capsules daily, and assessments of arterial blood gases (ABG) and dyspnea were performed every 4–8 weeks. The findings indicated that 40% of participants experienced significant improvements, with at least a 10 mmHg rise in arterial oxygen pressure (PO_2_) or a reduction in the alveolar-arterial gradient. Additionally, all six subjects who responded to garlic reported less exertional dyspnea (35). Likewise, children with hepatopulmonary syndrome received garlic powder capsules as part of a study to examine the effect of oral garlic on arterial oxygen pressure. Ten boys and five girls comprised the study cohort. They had a variety of underlying diseases, including presinusoidal portal hypertension, autoimmune hepatitis, cryptogenic cirrhosis, and biliary tract atresia. The findings showed that the mean arterial oxygen pressure (PaO_2_) increased by 10 mmHg in 53.3% of the patients, and PaO_2_ values of responders were significantly improved compared to those of non-responders. These results imply the effect of oral garlic supplements administration in children with hepatopulmonary syndrome on improving oxygenation and reducing dyspnea (36) (Table 1).

A trial was conducted on non-critically ill COVID-19 patients who received either Gallecina capsules or a placebo along with remdesivir for five days or until discharge. At discharge, parameters such as arterial oxygen saturation (SaO_2_) and respiratory symptoms were identical between groups, but body temperature was considerably lower in the Gallecina group. On days 3 and 4, as well as upon discharge, the Gallecina group required significantly less supplementary oxygen. Although there was no substantial improvement in clinical status by day 6, Gallecina did demonstrate a significant decrease in oxygen requirements on various days, indicating prospective advantages in COVID-19 patients who are not critically ill (37).

In a study of healthy, overweight adults without respiratory illnesses, the effects of exercise and garlic supplements on lung function were evaluated. Standard methods were used to measure peak expiratory flow rate (PEFR), FEV_1_, FVC, and Forced Expiratory Flow at 25-75% (FEF25-75%). Garlic consumption was found to substantially decrease FVC and FEV_1_, maintain PEFR, and significantly raise FEF25-75% (38).

The purpose of a study was to determine whether garlic powder may help reduce the signs and symptoms of pulmonary tuberculosis. Participants were divided into two groups: a treatment group and a control group. Tuberculosis signs and symptoms were monitored using observation sheets and standard operating procedures. Following the administration of garlic powder, statistical analysis showed a significant reduction in pulmonary tuberculosis signs and symptoms (including night sweats, cough with phlegm, fever, cough mixed with blood, chest pain, dyspnea, etc.). In contrast, there was no significant difference in tuberculosis symptoms in the control group (39).

Research on the effects of garlic supplements in various respiratory conditions has shown promising results. While these findings are promising, larger trials with more rigorous controls are needed to confirm these effects and further explore the specific mechanisms underlying the benefits of garlic in respiratory disorders. Future research should focus on identifying bioactive compounds in garlic, potential interactions with standard treatments, optimal dosages, and long-term effects for a comprehensive understanding of garlic supplementation in respiratory conditions.

## Carum copticum (L.) Benth. & Hook.f. (Ajowan)

*Carum copticum *(L.) Benth. & Hook.f. (*C. copticum*), commonly known as “Ajowan,” is grown around the world, particularly in places like Iran and several Indian states. In the past, *C. copticum* has been used for a variety of medical conditions, including abdominal tumors, bloating, diarrhea, decreased appetite, stomach discomfort, dyspnea, and fatigue (40). In addition to these applications, it has antifungal (41), anti-oxidant, immunomodulatory, anti-inflammatory (42), bronchodilatory (43), and antibacterial (44) properties.

### Effects of C. copticum extract

A study compared the bronchodilatory effects of a boiling extract of *C. copticum* with theophylline and a placebo in individuals with asthma. Specific airway conductance (sGaw) and PFT measures revealed that the *C. copticum* extract significantly raised PFT values over a range of time periods, with effects similar to those of theophylline but at lower concentrations. The bronchodilatory impact started after 30 min, peaked at 23 to 32% in PFTs between 90 and 120 min, and then began to decline after 150 min, which was similar to the theophylline effect. PFT results were not significantly altered by the placebo (45) (Table 1). These results highlight the potential therapeutic effects of *C. copticum* in treating asthma symptoms (including dyspnea) by indicating that it has a significant bronchodilatory impact on asthmatic airways comparable to theophylline at the concentrations examined.

## Crocus sativus L. (Saffron)

*C. sativus* is a perennial plant that is primarily grown in Iran and belongs to the Iridaceae family (46). In addition to being used as a food coloring and spice, saffron has been extensively utilized by traditional medicine as a stomachic agent, stimulant, nerve relaxant, expectorant, eupeptic, diaphoretic, anticatarrhal, carminative, antispasmodic, and aphrodisiac (47). It has also been indicated to have anti-oxidant (48, 49), anti-inflammatory and immunoregulatory (50), bronchodilatory (51, 52), antiapoptotic (53), autophagy regulatory (54), neuroprotective (55), renoprotective (47, 56), hepatoprotective (57), and hypnotic (58) properties. The subsequent section will delve into the impact of saffron on dyspnea. 

### Effects of C. sativus extracts

The effects of saffron supplementation on spirometry tests, high-sensitivity C-reactive protein (hs-CRP), and anti-heat shock protein 70 (anti-HSP70) were examined in a clinical experiment with patients who had mild to moderate allergic asthma. The patients were divided into two groups at random and treated for eight weeks. One group received saffron capsules daily, while the other group received a placebo. Spirometry parameters, hs-CRP, and anti-HSP70 levels were measured before and after the intervention. The findings showed that hs-CRP and anti-HSP70 concentrations were significantly lower in the saffron supplement group than in the placebo group. Furthermore, spirometry testing revealed notable improvements in the saffron group’s forced expiratory flow (FEF), FVC, FEV_1_, and FEV_1_/FVC ratio (59). It was investigated how saffron affected several parameters in patients with mild to moderate allergic asthma. Participants were randomized to either the control (placebo) group or the intervention (saffron) group. When compared to the placebo group, the results showed that patients in the saffron group experienced significant improvements in their clinical symptoms, including reduced use of inhaled salbutamol, decreased frequency of shortness of breath, improved sleep quality, and increased activity levels. Furthermore, the saffron group showed a significant reduction in the severity of their asthma. In addition, saffron supplementation significantly decreased triglycerides, low-density lipoprotein cholesterol, and systolic and diastolic blood pressure when compared to the placebo. Additionally, eosinophil and basophil counts of the saffron group exhibited a declining trend (60).

An investigation focused on using a neural network modified by a genetic algorithm to develop a clinical prediction system for assessing the effects of *C. sativus *supplements on individuals with allergic asthma. The model was intended to predict the effectiveness of saffron supplements on different asthma risk variables and the level of alleviation in asthma patients using data from men and women with mild to moderate allergic asthma. According to the results, the system performed exceptionally well, achieving an accuracy of over 99% for both the training and testing datasets. High accuracy levels were demonstrated by the neural network’s ability to forecast the influence on important factors such as hs-CRP, anti-heat shock protein, and spirometry metrics (FEV_1_/FVC ratio, FVC, FEV_1_, and FEF25%-75%) (61). 

### The effects of C. sativus constituents

*Crocin*

A clinical trial involving COPD patients with the following conditions: one- clinical criteria such as cough, sputum, shortness of breath, and two- spirometric findings FEV_1_/FVC < 70%, FEV_1_ < 80%, was designed to assess the impact of crocin supplementation on various parameters. A control group that received a placebo was compared with the intervention group that received crocin. The findings showed that crocin supplementation increased total anti-oxidant capacity while decreasing blood levels of nuclear factor kappa B (NF-κB) and total oxidant status. According to the 6-minute walking distance test (6MWD), patients also demonstrated greater exercise ability (62).

A study aimed to assess the preventative effects of crocin supplementation on interleukin (IL)-6, tumor necrosis factor-alpha (TNF-α), exercise capacity, and PFTs in COPD patients (with chronic dyspnea). The patients were divided between an intervention group that received crocin and a placebo group. By raising blood IL-6 levels, crocin supplementation had preventative effects and led to notable improvements in PFTs and 6MWD as compared to the placebo group. Additionally, toward the end of the research, crocin intervention resulted in a significant decrease in serum TNF-α levels (63).

In the context of dyspnea, studies on saffron and crocin supplementation showed potential results for respiratory diseases. Saffron decreased symptoms and asthma severity in allergic asthma patients, which influenced spirometry metrics. Crocin increased anti-oxidants, reduced inflammation, and improved exercise in dyspneic COPD patients, demonstrating anti-inflammatory properties while enhancing pulmonary and exercise function. These data highlight the efficacy of saffron and crocin as adjuvant treatments for addressing dyspnea-related respiratory diseases. Future research should prioritize large-scale randomized controlled trials to validate these findings, explore the underlying mechanisms of saffron and crocin effects, and investigate the therapeutic efficacy of crocin in treating dyspnea in patients. Furthermore, examining the synergistic effects of existing medications with saffron/crocin, as well as formulation strategies to improve absorption, could present new possibilities for optimizing treatment outcomes in respiratory disorders associated with dyspnea. Furthermore, additional research into neural network modeling for predicting therapy responses in dyspnea has the potential to enhance individualized respiratory care.

## Curcuma longa L. (Turmeric)

Turmeric belongs to the Zingiberaceae (ginger) family and is an evergreen herbaceous plant. It is widely grown throughout Asia. The rhizome, a thick, meaty underground stem encircled by the bases of old leaves, is a component of turmeric that may have therapeutic use. The turmeric plant has long been used in traditional medicine as a natural antiseptic, disinfectant, anti-inflammatory, and painkiller. It is also frequently used to heal skin irritations, promote gut flora, and aid with digestion. Additionally, it has been used as an easily accessible antiseptic for burns, bruises, and cuts throughout South Asia (64). Pharmacological investigations unveiled its anti-inflammatory (65), anti-oxidant (66), antiproliferative (67), antiasthmatic (68), immunomodulatory (69, 70), antiallergic (71), bronchodilatory (72), and antidote (73) properties. The effect of *Curcuma longa* (*C. longa*) on dyspnea will be covered in detail in the next section.

### Effects of C. longa constituents

*Curcumin*

Patients with mild to moderate bronchial asthma were evaluated for the safety and effectiveness of curcumin as a supplementary treatment. Individuals were randomly assigned to one of two groups, with Group A receiving regular asthma treatment and Group B receiving the same treatment plus daily curcumin for a month. Curcumin supplements significantly improved mean FEV_1_ values, suggesting the bronchodilatory effect and its effect on dyspnea (74). This effect was also supported by the findings of an experimental study (75). 

The effects of curcumin on patients with chronic bronchial asthma were examined. Obesity and asthma frequently coexist because of systemic inflammation and airway restrictions. Patients were divided into two groups: one group received standard asthma treatment alone, while the other group received the same treatment plus curcumin for two months. The results showed that the FEV_1_ and FEV_1_/FVC ratio of the curcumin group had significantly increased. After therapy, curcumin supplementation resulted in a drop in the neutrophil count but no change in the total white blood cell count. Body mass index and leptin levels, however, did not significantly differ between the two groups (76).

It was investigated whether curcumin supplements were beneficial for those with chronic bronchial asthma. Two groups of patients with a diagnosis of chronic bronchial asthma were established. Group 2 received the same treatment plus curcumin, whereas group 1 received standard asthma treatment. FEV_1_ level and the percentage of predicted FEV_1_ did not substantially differ between groups at the beginning of the study or two months later. Following treatment, FEV_1_ increased significantly in group 1 and highly significantly in group 2. The value of FVC was considerably higher in both groups after treatment. There were no apparent group variations in the FVC values. After therapy, the FVC and the FEV_1_/FVC ratio of group 2 were significantly increased. Although there was no significant difference between the groups, the peak expiratory flow (PEF) value rose considerably in both groups after treatment. Furthermore, IL-6 levels in group 2 patients were significantly lower than those of group 1 patients. Nevertheless, following the supplement, the superoxide dismutase (SOD) level in neither group changed significantly (77).

Curcuminoids, which include bisdemethoxycurcumin, curcumin, and demethoxycurcumin, were found to have significant clinical effects on markers of pulmonary function and systemic inflammation in a randomized double-blind placebo-controlled pilot study that involved male subjects with chronic sulfur mustard-induced pulmonary complications. For four weeks, the subjects received either a placebo or curcuminoids. The FEV_1_/FVC ratio improved more with curcuminoids than with placebo, even though FEV_1_ and FVC stayed similar between groups. The modulation of several inflammatory mediators, such as IL-6 and IL-8, TNF-α, transforming growth factor-β (TGF-β), hs-CRP, calcitonin gene-related peptide (CGRP), substance P, and monocyte chemotactic protein-1, was also considerably more affected with curcuminoids than with placebo (78).

A clinical trial was designed to find out how nanocurcumin affected the inflammatory cytokines in COVID-19. In December 2019, the coronavirus, commonly referred to as COVID-19, in Wuhan, China, became a global epidemic and public health emergency with a high rate of morbidity and fatality (79). IL-1β, IL-6, TNF-α, and IL-18 levels were significantly higher in COVID-19 patients than in healthy controls. Following nano-curcumin administration, IL-1β gene expression and secretion levels, as well as IL-6 expression and secretion in serum and supernatant, significantly decreased. Nevertheless, neither TNF-α levels nor IL-18 messenger ribonucleic acid (mRNA) expression was impacted by nano-curcumin. Fever, cough, and dyspnea were among almost every clinical manifestation that showed substantial improvement in the nano-curcumin group following treatment (80).

The effectiveness and safety of an oral nano-curcumin formulation were evaluated in outpatient COVID-19 patients with mild to moderate symptoms in a triple-blind, randomized, placebo-controlled clinical trial. The formulation of nanocurcumin or a placebo was administered to patients at random. All symptoms (including dyspnea) improved faster in the therapy group, except for sore throat, with significant improvements in taste and smell disturbances, cough, and chills. Furthermore, the treatment group had lower CRP serum levels and considerably greater lymphocyte counts than the group receiving a placebo at the end of the trial (81).

In moderately obese menopausal women, the effects of moderate-volume-high-intensity interval training with nano-curcumin supplementation, low-volume high-intensity interval training, and moderate-volume high-intensity interval training were compared with respect to physical performance, muscular fitness, respiratory function, and cardiovascular hemodynamics. After eight weeks, the results demonstrated that moderate-volume-high-intensity interval training and low-volume-high-intensity interval training therapies significantly increased oxygen pulse and VO_2_max. Muscular fitness and physical performance metrics, such as running distance, sit-to-stand test, muscle quality index, quadriceps strength, and hand grip strength, improved with moderate-volume-high-intensity interval training, both with and without nano-curcumin, as compared to baseline. In contrast to low-volume-high-intensity interval training, moderate-volume-high-intensity interval training demonstrated increased FVC. According to the study, moderate-volume-high-intensity interval training, particularly when combined with nano-curcumin supplementation, is a safe and efficient method for improving physical performance and cardiorespiratory fitness in obese menopausal women. Both low-volume-high-intensity interval training and moderate-volume-high-intensity interval training can be time-efficient methods to improve these outcomes (82).

The frequency of curcumin-rich curry consumption and a number of health outcomes were investigated over an average follow-up time of 11.6 years in a prospective cohort study. According to the study, consuming more curcumin-rich curry showed a beneficial correlation with certain health indicators. It is interesting to note that there were non-linear correlations with variables like COPD prevalence and FEV_1_/height^2^ (83) (Table 2).

Several major findings emerged from clinical trials that investigated the effects of curcumin on respiratory diseases, with a specific emphasis on dyspnea. Curcumin supplementation was found to significantly improve lung function indices such as FEV_1_ and FEV_1_/FVC ratio, indicating reduced airway obstruction and increased respiratory efficiency in individuals with bronchial asthma and pulmonary complications. Notably, in COVID-19 patients, nano-curcumin resulted in significant reductions in dyspnea as well as lower levels of inflammatory cytokines. Furthermore, curcumin administration in chronic bronchial asthma patients enhanced FEV_1_ levels and alleviated dyspnea. These findings support the ability of curcumin to manage dyspnea and improve respiratory function in a variety of respiratory diseases. 

When discussing the characteristics of these studies, the inclusion of placebo-controlled designs and objective outcome measures provides strong evidence of curcumin’s efficacy in treating dyspnea and related respiratory symptoms. However, limitations such as varying doses, sample sizes, and treatment periods between studies make direct comparisons and generalization difficult. Future research directions may include standardizing curcumin formulations, investigating long-term safety profiles, developing personalized dosing strategies based on dyspnea severity, and elucidating the molecular mechanisms underlying the beneficial effects of curcumin on dyspnea in respiratory-related diseases. Furthermore, novel scientific concepts may include evaluating the impact of curcumin on dyspnea-specific quality-of-life metrics, conducting larger-scale trials in diverse patient populations, and investigating synergies between curcumin and traditional respiratory treatments to optimize dyspnea management and improve respiratory health outcomes.

## Eucalyptus globulus Labill. (Eucalyptus)

The enormous and durable *Eucalyptus globulus* Labill. (*E. globulus*) tree, also referred to as southern blue gum, was discovered for the first time on the island of Tasmania in 1792. It belongs to the Myrtaceae family, one of the larger genera (84). Anti-oxidant, antimicrobial (85), analgesic (86), anti-inflammatory (87), and bronchodilator (88) properties have been demonstrated for the *E. globulus*. The following section will provide more detailed information on how *E. globulus* affects dyspnea.

### Effects of E. globulus extracts

In a study, the effects of eucalyptus aromatherapy oil on anxiety, back pain, and dyspnea in patients with post-COVID syndrome—a condition marked by persistent symptoms that remain for more than 12 weeks after infection and have no other explanation—were examined. Using eucalyptus oil, anxiety, back discomfort, and dyspnea all considerably decreased (89).

The effects of nebulized eucalyptus on arterial blood gases and physiological markers in patients with mechanical ventilation were explored. Patients were divided into control and nebulized eucalyptus groups. While the control group received saline alone, the nebulized eucalyptus group was administered eucalyptus diluted in saline. The Glasgow Coma Scale, arterial blood gas levels, and ventilator settings were tracked. After three days, the nebulized eucalyptus group PaO_2_, SaO_2_ increased, and peak inspiratory pressure (PIP) significantly decreased, although the results initially showed no differences between the groups (90).

### Effects of E. globulus constituents

*Cineole (Eucalyptole)*

The effects of cineole were examined in a multicenter, double-blind, placebo-controlled study with individuals who had stable COPD. During the winter, patients were randomized to receive either cineole or a placebo. Cineole significantly decreased the primary outcomes, which included the severity, duration, and frequency of exacerbations. These findings were further confirmed by secondary outcomes, such as improvements in dyspnea, lung function, and quality of life (91). Cineole was used as a concurrent treatment for asthma patients in a double-blind, placebo-controlled multicenter study. Cineole or a placebo was administered to the patients. When compared to the placebo group, the cineole-treated group showed a substantial improvement in the primary outcome measures, which evaluated lung function, asthma symptoms, and quality of life. The Asthma Quality of Life Questionnaire, FEV_1_, and asthma symptoms were among the individual outcome measures that also demonstrated statistical significance (92) (Table 2).

A series of investigations on the medicinal potential of eucalyptus and its ingredient, cineole, revealed promising results. These data demonstrate the beneficial effects of eucalyptus and cineole in controlling respiratory symptoms, probably through their mucolytic, broncho-dilating, and anti-inflammatory properties. While the studies employed strong methodologies, future research may investigate optimal dosages, long-term effects, and underlying mechanisms to enhance the understanding and application of eucalyptus-based therapies for a broader range of respiratory conditions.

## Mentha × piperita L. (Peppermint)

Native to Europe, *M. piperita* is an herbaceous, aromatic perennial that is grown in northern Asia, Canada, North Africa, the United States, and many other countries (93). Numerous pharmacological effects, including antiallergic (94), antibacterial (95), anticancer (96), anti-inflammatory (97), anti-oxidant (98), and antiasthmatic (99), are present in its extracts and essential oil. In the following section, a detailed exploration of the impact of *M. piperita* on dyspnea will be provided.

### Effects of M. piperita essential oil

The effect of consuming peppermint essential oil on the physiological parameters and exercise performance of healthy male students over a ten-day period was examined by supplementing mineral water + peppermint essential oil. Blood pressure, heart rate, and spirometry measures like FVC, PEF, and peak inspiratory flow (PIF) were measured before and after the supplementation period. A treadmill-based exercise test utilizing the Bruce protocol with metabolic gas measurement was provided to the individuals. PEF, FVC, PIF, exercise performance measures (power, work, and time to exhaustion), and respiratory gas analyzer parameters (VO_2_ and VCO_2_) all showed notable improvements following supplementation (100).

In a randomized crossover design, healthy subjects completed a graded maximal activity test ten days after consuming either peppermint essential oil or a control. For expired gas characteristics and performance metrics, there was no considerable difference between the control and peppermint essential oil trials. Likewise, resting cardiopulmonary parameters did not alter from one visit to the next (101) (Table 3).

## Effects of M. piperita constituents

### L-menthol

A study intended to determine how L-menthol nasal inhalation affected healthy people’s ventilation and respiratory discomfort during loaded breathing. When breathing through devices with flow-resistive and elastic loads, participants used a visual analog scale (VAS) to score their level of respiratory discomfort. During both forms of loading, inhaling L-menthol significantly reduced respiratory discomfort, as evidenced by a notable drop in VAS values. Without changing ventilation or breathing patterns, this effect was noted. When flow-resistive loading was compared to elastic loading, the VAS ratings decreased more noticeably during the former. Inhaling air with a strawberry taste had no effect on ventilation, breathing patterns, or VAS scores, indicating that smell was not a factor in the alleviation of respiratory discomfort (102).

The impact of inhaling a 1% L-menthol solution as a premedication for fiberoptic bronchoscopy was examined in relation to the frequency of cough and the irritation of the tracheobronchial mucosa in a blinded, randomized, and placebo-controlled trial. For the standard scent, the placebo group received 0.05% L-menthol, while the verum group was administered 1% l-menthol solution. PEF was measured both before and after inhalation, bronchoscopists evaluated mucosal irritation, and cough frequency was monitored during fiberoptic bronchoscopy. With no significant difference between groups, patients reported less coughing and dyspnea after undergoing fiberoptic bronchoscopy compared to before. Although the tolerability of fiberoptic bronchoscopy did not improve, 1% L-menthol inhalation significantly raised PEF (103). 

The effects of olfactory stimulation by L-menthol on the neural respiratory drive and different dimensions of dyspnea caused by inspiratory resistive loaded breathing in patients with COPD were investigated. An L-menthol-scented patch was utilized to deliver L-menthol, and a strawberry-scented patch served as the placebo. Breathing through inspiratory resistance caused dyspnea, and the multidimensional dyspnea profile was used to measure the condition. L-menthol considerably reduced air hunger, anxiety, breathing discomfort, fear, and physical and mental breathing effort in COPD patients during inspiratory resistive loaded breathing; however, it did not affect neural respiratory drive or breathing patterns. L-menthol did not considerably enhance the affective aspect of dyspnea in the control group despite lowering air hunger, unpleasantness, and mental breathing effort (104).

The effect of L-menthol olfactory stimulation in reducing exertional dyspnea in patients suffering from chronic breathlessness syndrome was examined. Two groups, A and B, participated in the 6MWT. Group B performed the tests in reverse order, with group A using a surgical mask (placebo) for the first test and L-menthol for the second. According to the results, dyspnea in group A during the second 6MWT under the L-menthol condition significantly decreased. Furthermore, there was a significant difference in the modified Borg scale gain between the L-menthol and placebo treatments (105).

The effect of administering L-menthol on the endurance exercise capacity of male runners was evaluated. Participants underwent three distinct trials—water intake, L-menthol mouth rinse, and L-menthol ingestion—in random sequences every five min while running on treadmills at fixed intensities of their anaerobic thresholds until exhaustion. Before and after running, the dyspnea threshold against external inspiratory resistance was measured, and breathing comfort was evaluated after fluid intake. The results demonstrated a large effect size and a significantly longer running time when L-menthol was consumed as opposed to water. At exhaustion, breathing comfort was much higher when L-menthol was consumed than when water was consumed. Following a run, the dyspnea threshold dropped when water was consumed, but it stayed high when mouthwash and L-menthol were consumed, suggesting a significant difference. Consuming L-menthol improved running-related breathing comfort, increased endurance capacity, and maintained the dyspnea threshold against external inspiratory resistance after intense running, indicating that L-menthol may help endurance athletes perform better during exercise and have more comfortable breathing (106).

Research on peppermint essential oil and L-menthol yielded interesting findings regarding their effects on respiratory function and exercise performance. The use of peppermint essential oil improved pulmonary function and exercise performance in healthy individuals, while L-menthol inhalation significantly reduced respiratory discomfort during loaded breathing without affecting ventilation. L-menthol has also been shown to reduce cough frequency, increase PEF, and alleviate dyspnea in individuals with COPD and chronic breathlessness syndrome. Furthermore, L-menthol consumption improved running-related breathing comfort, endurance capacity, and the dyspnea threshold in male runners during intense activity. These findings indicate that both peppermint essential oil and L-menthol may influence respiratory sensations via sensory processes. While the studies had strengths, such as controlled designs and a wide range of outcome measures, shortcomings, such as limited sample sizes and short-term assessments, were identified. Future plans include performing large-scale clinical trials to validate these effects, further researching the underlying mechanisms, and determining ideal dosages and delivery methods for future therapeutic applications in respiratory health and exercise physiology.

### Nigella sativa L. (Black seed)

*Nigella sativa *L. (*N. sativa*), a spicy seed from the Ranunculaceae family, is often known as black seed or black cumin. This multipurpose seed has been used for millennia in China, Syria, Turkey, Pakistan, and India as a natural cure for illnesses such as depression, heart disorders, hepatotoxicity, neurotoxicity, and renal toxicity, in addition to being a spice and food preservative. Thymoquinone is a major component in *N. sativa* (107). Modern pharmacological investigations have illustrated its anti-inflammatory (108), anti-oxidant (109), antiasthmatic (110), bronchodilatory (111), antiallergic, and immunomodulatory (112) properties. Detailed coverage of the impact of *N. sativa* on dyspnea will be provided in the following section.

### Effects of N. sativa extracts and essential oil

A case report described a rare incidence of exogenous lipoid pneumonia caused by consuming *N. sativa* seed oil. The patient, a 50-year-old man, reported consuming *N. sativa* oil for tonification for an extended period (8 bottles of 500 ml, 8 months). He had a history of persistent coughing, sputum production, and worsened dyspnea upon exertion. Routine blood tests and cultures revealed no abnormalities, even though the physical examination showed bilateral lung crackles; the imaging investigations indicated honeycomb fibrosis, bilateral basal interstitial disease, and thoracic distension, and the lung function tests were normal. A blackish lavage fluid with fat globules, suggestive of lipid aspiration, and a high macrophage count were seen during bronchoscopy (113). This instance highlights the importance of diagnosing exogenous lipoid pneumonia in patients with atypical respiratory symptoms and considering uncommon causes, such as the use of *N. sativa* oil.

A clinical trial investigated the effect of boiling the extract of *N. sativa* on adult patients’ asthma over the course of three months. Patients were randomized to receive the *N. sativa* extract in the study group and a placebo solution in the control group. In comparison to the first visit, asthma symptoms (e.g., coughing, wheezing, and tightness) in the study group showed significant improvements in symptom frequency, wheeze, and PFT values throughout the second and third visits, with additional improvements noted at the third visit. By the end of the trial, every symptom in the study group was noticeably better than those in the control group. Additionally, whereas medication use remained constant in the control group, it declined in the research group (114) (Table 4).

An investigation was conducted into the bronchodilatory effects of boiled extract of *N. sativa* on asthmatic patients’ airways. The extract significantly raised all measured PFTs over the majority of periods. However, compared to theophylline, the increase in FEV_1_, maximal mid-expiratory flow (MMEF), and maximal expiratory flow (MEF_50_) produced by both dosages of the boiling extract was much smaller. Additionally, the increase in MEF_75_ and MEF_25_ caused by the lower extract dosages was much less than that caused by theophylline. The bronchodilatory effect of the extract began at 30 min, similar to that of theophylline, and decreased 150 min after administration, just as theophylline did. Furthermore, at 30 min after administration, the effects of both extract dosages were noticeably less noticeable than those of salbutamol (115).

In an investigation, the broncho-relaxant effects of *N. sativa* were assessed in individuals with chronic bronchial asthma. Patients received *N. sativa* after clinical evaluations, pulmonary function testing, and serum electrolyte studies. The results showed that this plant significantly reduced asthma attacks and improved FEV_1_ and FVC compared to the control group (116).

The effectiveness of *N. sativa* oil supplementation on clinical and inflammatory markers in asthmatic patients was examined. The *N. sativa* oil group showed a substantial decrease in blood eosinophils and a significant improvement in the mean score on the Asthma Control Test (ACT) when compared to the placebo group. With *N. sativa* oil supplementation, there was a positive trend toward improvement in FEV_1_ (117). 

It was investigated how *N. sativa* supplementation affected airway inflammation and airflow restriction in patients with partially controlled asthma. In addition to maintenance inhalation medication, the *N. sativa*-1 and *N. sativa*-2 groups were administered 1 and 2 g/day of *N. sativa*, respectively, whereas the control group received a placebo. The *N. sativa*-2 group showed significant increases in FEV_1_ and FEF_25-75_ at 6 and 12 weeks. At 6 and 12 weeks, PEF variability in the *N. sativa*-1 and *N. sativa*-2 groups was significantly lower than in the controls. After 12 weeks, both *N. sativa* groups showed a significant drop in blood immunoglobulin E (IgE) and fractional exhaled nitric oxide (FeNO) levels. Furthermore, both *N. sativa* dosages significantly improved the ACT score at 6 and 12 weeks and raised blood interferon-gamma (IFN-γ) levels at 12 weeks. There were noticeably fewer exacerbations in the *N. sativa*-1 group (118). 

The preventive effects of a boiling aqueous extract of *N. sativa* seed on chemical war victims with respiratory symptoms, comprising breathlessness, cough, wheezing, and chest tightness, were examined. The victims were divided into study and control groups, and each day, they were given either a placebo or the extract. PFTs, wheezing, and respiratory symptoms were evaluated three times. When compared to the first visit, the symptoms of the study group and PFT levels significantly improved at the second and third visits, with even more improvements noted at the third. There were significant differences between the study and control groups, with the study group showing better results (119). 

In patients with mild to moderate COPD with symptoms such as coughing, exercise intolerance, shortness of breath, and wheezing, the possible advantages of *N. sativa* oil supplementation on PFTs, inflammation, and oxidant-anti-oxidant markers were examined. Patients were randomized to either the *N. sativa* oil group, which received additional *N. sativa* oil, or the control group, which received regular medication alone. Anti-oxidants such as catalase (CAT), glutathione peroxidase (GPx), glutathione (GSH), SOD, vitamin C, and E were significantly higher in the *N. sativa* oil group, while oxidative and inflammatory markers like IL-6, protein carbonyl, thiobarbituric acid reactive-substances (TBARS), and TNF-α were much lower. Furthermore, when comparing the *N. sativa* oil group to the control group and baseline levels, notable improvements in PFTs were reported (120). 

The purpose of a study was to investigate how *N. sativa* (Baraka®, Pharco Pharmaceuticals, Cairo, Egypt) and vitamin D3 (Davalindi®, Medical Union Pharma, Cairo, Egypt) alone and combined supplemental treatments affected the viral clearance and symptom relief of COVID-19 patients throughout a 14-day follow-up period. Four groups were randomly assigned to mild to moderately ill COVID-19 patients: one group received *N. sativa*, another received vitamin D3, a third received both supplements, and a control group. The *N. sativa*-vitamin D3 combination, when used in conjunction with standard COVID-19 therapy, was found to reduce symptoms and speed up viral clearance considerably. Within days after starting treatment, patients in this combination treatment group notably alleviated their cough, dyspnea, fatigue, headache, and rhinorrhea. Additionally, on days 7 and 14, the combination group outperformed the other groups in terms of negative polymerase chain reaction (PCR) test findings (121).

Thymoquinone investigations in rats demonstrated harmful effects (including symptoms such as diarrhea, dyspnea, and hypo-activity) after oral doses, whereas an unusual case report emphasized exogenous lipoid pneumonia caused by a patient’s excessive ingestion of *N. sativa* seed oil revealing symptoms like persistent coughing, worsened dyspnea upon exertion, and bilateral lung abnormalities. Clinical research on *N. sativa*, on the other hand, has produced consistently favorable results in terms of controlling dyspnea, reducing asthma symptoms, lowering drug dependence, and demonstrating preventive and bronchodilatory effects in various patient groups. Despite these hopeful findings, constraints such as small sample sizes, limited follow-up periods, and potential biases in study design may limit the generalizability of the results. Future research could focus on optimizing *N. sativa* dosages, investigating its long-term effects, elucidating its mechanisms of action in respiratory diseases, and examining potential interactions with existing asthma medications to enhance patient care and outcomes.

## Rosmarinus officinalis L. (Rosemary)

*Rosmarinus officinalis *L. (*R. officinalis*), the scientific name for rosemary, is a perennial plant belonging to the Lamiaceae family. Traditional medicine has long used rosemary to treat a wide range of illnesses, such as rheumatic pain, depression, gastrointestinal disorders, epilepsy, headaches, hysteria, exhaustion, anxiety, pain management, spasms, stomachaches, and respiratory disorders (122). In addition, physio-pharmacological investigations revealed its antiapoptotic (123), anti-inflammatory (124, 125), antidote (126, 127), anti-obesity (128), cardioprotective (129, 130), antirheumatic (131), antinociceptive (132), antidepressant (133), bronchodilatory (134), and neuroprotective (135, 136) properties. The subsequent section will delve into a thorough analysis of the influence of *R. officinalis* on dyspnea.

### Effects of R. officinalis extracts

In a study evaluating the effect of leaf extract on asthmatic patients who were not responding to conventional therapies, clinical symptoms (including coughing and dyspnea), spirometry data, exhaled nitric oxide levels, and ACT scores were evaluated. *R. officinalis* ameliorated clinical symptoms such as coughing, sputum production, and wheezing. The study found that ACT scores and nitric oxide levels in the exhaled air were significantly improved (137).

The effects of rosemary hydroalcoholic extract on the activities of daily living (ADLs) of patients with COPD were examined. While the control group received placebo capsules, the intervention group was administered rosemary capsules. Before and after the intervention, ADLs were assessed using the Lawton Instrumental Activities of Daily Living (IADL) and the London Chest Activity of Daily Living scale (LCADL). Regarding changes in ADL scores, there were no appreciable variations between the two groups. It is interesting to note that the intervention group outperformed the control group in terms of gains in IADL after controlling for obstructive sleep apnea (OSA) (138).

### Effects of R. officinalis constituents

*1,8-cineol (Eucalyptole)*

In a study, patients with steroid-dependent bronchial asthma were randomized to receive either 1,8-cineol or a placebo. Every three weeks, the dosage of oral glucocorticosteroids was progressively decreased. The study demonstrated the steroid-sparing effect of 1,8-cineol by showing a substantial decrease in the daily prednisolone dosage in the active treatment group compared to the placebo group. Reducing prednisone by 2.5 mg every three weeks did not have a significant effect on PEFR or lung function in the active therapy group, nor did it increase the regular use of salbutamol. However, following the first prednisolone reduction, PEFR considerably decreased in the placebo group, which resulted in a large rise in the usage of salbutamol. After the 2.5 mg prednisolone reduction, the placebo group showed noticeably greater dyspnea scores than the active treatment group, even though dyspnea scores did not differ substantially between the groups before the glucocorticosteroid reduction (139) (Table 4). 

The examined studies on herbal interventions in dyspnea management offer valuable insights. Rosemary leaf extract demonstrated efficacy in alleviating asthma symptoms, including dyspnea, while also improving ACT scores and reducing exhaled nitric oxide levels. In COPD patients, rosemary hydroalcoholic extract did not notably affect activities of daily living but showed potential benefits in instrumental activities of daily living, which could indirectly impact dyspnea. Additionally, 1,8-cineol displayed a steroid-sparing effect in severe asthma, reducing prednisolone dosage without exacerbating dyspnea or compromising lung function. These findings suggest that herbal remedies, such as rosemary and 1,8-cineol, may hold promise in managing dyspnea associated with respiratory conditions. Future well-controlled trials should further investigate their mechanisms of action and long-term effects on dyspnea to establish their role in dyspnea management strategies.

## Thymus vulgaris L. (Thyme)

*Thymus vulgaris* L. (*T. vulgaris*), thyme, also called “garden thyme,” is a fragrant perennial blooming plant that is a member of the *Lamiaceae* family. Despite being indigenous to Southern Europe, *T. vulgaris* is reported to be found worldwide. Due to its antibacterial and therapeutic effects, the herb was mainly used to treat wounds. Ancient Europeans used its aerial parts for fumigation and to cure skin and respiratory illnesses, demonstrating the anti-infective properties of the plant (140). Current research has also confirmed its pharmacological effects, including anti-inflammatory, anti-oxidant (141), immunomodulatory (142), bronchodilatory (143), antimicrobial (144), and antitumor (145) properties. The effects of *T. vulgaris *on dyspnea are discussed in the next section.

### Effects of T. vulgaris powder and essential oil

A study examined how *T. vulgaris *influenced coughing in children with mild to moderate asthma exacerbations aged 5 to 12 years. Participants were divided into control and intervention groups. While the control group received standard medical treatment along with a placebo syrup, the intervention group received *T. vulgaris* powder in addition to standard medical care. Activity-induced cough was significantly reduced after the intervention, and a statistically significant difference was observed between the groups. However, there was no statistically significant decrease in wheezing or dyspnea. While FEV_1_/FVC, PEF, and FEF_25-75_ of the vital capacity (FEF25-75%) did not show significant variations between the groups, spirometry data showed a significant difference in FEV_1_ after the intervention (146).

The purpose of a study was to evaluate how thyme oil aromatherapy affected the hemodynamic markers, vital signs, and symptoms of COVID-19 patients. While the control group received standard medical treatment, patients in the experimental group inhaled thyme oil. Findings showed that thyme oil significantly alleviated symptoms such as diarrhea, dizziness, cough, headache, loss of appetite, muscle and joint pain, secretion, shortness of breath, and weakness. Although there were improvements in runny nose, nausea and vomiting, and loss of taste and smell, these modifications were not of statistical significance. Thyme oil had a significant favorable effect on pH management and CO_2_ levels, and it also raised SaPO_2_, maintained blood pressure, and significantly decreased respiratory rate, pulse rate, and body temperature (147).

In brief, *T. vulgaris* showed potential in reducing cough and improving lung function in children with asthma. At the same time, thyme oil aromatherapy demonstrated probable advantages in alleviating symptoms and stabilizing vital signs in COVID-19 patients. Further research is warranted to explore the mechanisms of action and optimize the use of these interventions for respiratory conditions.

## Zataria multiflora Boiss.

*Zataria multiflora *Boiss. (*Z. multiflora*), a thyme-like plant in the *Lamiaceae* family grows wild only in central Afghanistan, southern Iran, and Pakistan. The aerial parts of *Z. multiflora* are not only a popular culinary herb, but they are also used in traditional medicine for their antidiarrhea, anthelmintic, carminative, analgesic, and antibacterial effects (148). Recent pharmacological studies have highlighted other biological characteristics of this herb, including antiasthmatic (16), neuroprotective (149, 150), anti-oxidant, antibacterial (151), spasmolytic (152), bronchodilatory (153), and anti-inflammatory (154) properties. The following section discusses the effects of *Z. multiflora* on dyspnea.

### The effect of Z. multiflora extracts

In a study, the effects of *Z. multiflora* on wheezing in asthmatic patients, FEV_1_, and plasma nitrite levels were examined. Three groups of asthmatic patients were randomly assigned: two treatment groups that received varying dosages of *Z. multiflora* and a placebo group. Comparing the *Z. multiflora*-treated groups to the baseline, the results showed a significant decrease in daytime and exercise-related wheeze. Furthermore, following treatment, nitrite levels dramatically dropped in the *Z. multiflora* high-dose group, while FEV_1_ markedly increased in these groups. On the other hand, during the course of the study, the examined parameters showed very little change in the placebo group (155).

The effects of *Z. multiflora* on oxidative stress, cytokine levels, PFTs, and clinical symptoms (chest wheeze, night cough, and wheeze) in asthmatic patients were examined. Three groups of asthmatic patients were studied: two groups received *Z. multiflora* extract treatment, and the third group received a placebo. When compared to baseline, the clinical symptoms and PFTs of the *Z. multiflora* groups at months one and two showed notable improvements. Furthermore, following treatment with both doses of the extract at month two, improvements in oxidative stress, cytokine levels, and gene expression were noted in comparison to baseline (156).

The purpose of a study was to evaluate the bronchodilatory effect of a *Z. multiflora* hydro-ethanolic extract in individuals with asthma. PFTs were performed before and at different intervals (15, 30, 60, 90, 120, 150, and 180 min) following the administration of the extract to patients with asthma and theophylline syrup to a few other patients. According to the primary outcome measures, the *Z. multiflora* extract considerably raised all PFT values between 30 and 180 min after treatment, which was comparable to the effect of theophylline. The effects of theophylline decreased 150 min after administration, whereas the extract-enhanced PFT values sharply dropped 180 min later. After administering the medication, there was no significant difference in PFT values between the extract and theophylline at baseline, 30 min, or 180 min (153). The bronchodilatory effect of the plant indicated in the above study was supported by an experimental study showing the relaxant effect of the plant on tracheal smooth muscle (16, 157-159).

The purpose of a research was to find out how *Z. multiflora* affected PFTs in veterans who had been exposed to sulfur mustard gas 27–30 years earlier. Veterans were allocated into two groups, each receiving varying dosages of *Z. multiflora* and a placebo group. MDA levels dropped in the treated groups, also total and other white blood cell counts significantly decreased. Anti-oxidant markers such as thiol, SOD, and CAT also significantly increased. The treated groups showed a significant improvement in PFTs, such as FVC and PEF, as compared to the placebo group (160).

Moreover, significant improvements in respiratory symptoms and serum cytokine levels were noted in a study examining the effects of *Z. multiflora* extract on veterans exposed to sulfur mustard gas more than 20 years ago. Patients were divided into two treatment groups, each receiving varying doses of Z*. multiflora* extract, and a placebo group. In both trial phases, the treated groups showed elevated FEV_1_ values, along with notable improvements in respiratory symptoms, including nighttime cough, exercise-induced cough, and chest wheezing. Additionally, compared to the placebo group, the treated groups’ serum levels of inflammatory markers such as epidermal growth factor (EGF), monocyte chemotactic protein 1 (MCP-1), TNF-α, and vascular endothelial growth factor (VEGF) substantially decreased (161).

A study examined the effect of *Z. multiflora* in patients who had been exposed to sulfur mustard for a long time. Participants were separated into three experimental groups, a placebo group, and two other groups, each receiving different doses of *Z. multiflora* extracts and a placebo group at random. The findings showed that, in comparison to the baseline, serum levels of IL-2, IL-6, and IL-8 dropped, whereas levels of IL-10 and IFN-γ significantly rose in the treatment groups. PFT indices such as MMEF and MEF25, 50, and 75 also demonstrated notable improvements in the treatment groups (162) (Table 5).

The purpose of a study was to evaluate how *Z. multiflora* affected the clinical symptoms, PFTs, oxidative stress, and CRP levels of COPD patients. Three groups of patients were formed, two of which received *Z. multiflora* extract and one of which received a placebo. In comparison to baseline values, the results showed significant improvements in sputum production in the higher *Z. multiflora* group and clinical symptoms, including chest wheezing and dyspnea, in the *Z. multiflora* groups at 1 and 2 months after treatment. Furthermore, following two months of treatment with *Z. multiflora* groups, FEV_1_ demonstrated a notable improvement. After two months of medication, the levels of malondialdehyde (MDA) and nitrite were considerably lower than baseline in the higher *Z. multiflora* group. The thiol contents and the SOD and CAT activities in the higher *Z. multiflora* group in both extract-treated groups significantly rose at month two in comparison to baseline, as well as CRP levels in both treated groups significantly decreased at the end of the trial in comparison to baseline (163). The effects of *Z. multiflora* on asthmatic patients described in the above clinical studies are supported by various experimental results indicating the effects of the plant on an animal model of asthma (164-168).

Furthermore, the effects of *Z. multiflora* extract on respiratory symptoms, PFTs, and inflammatory cytokines in patients with COPD were examined. Three groups of COPD patients were established: two groups received varying doses of *Z. multiflora* extract, and the third group received a placebo. Serum levels of TNF-α and IL-8 were found to have significantly decreased following two months of extract treatment. PFT parameters, such as FVC and FEV1, also showed improvements during the course of treatment. Following a month of treatment with the higher dose, PEF increased noticeably. Following one and two months of treatment with both doses of *Z. multiflora*, there was a noticeable amelioration in respiratory symptoms such as cough, chest tightness, and dyspnea as compared to baseline values (169).

Another clinical trial evaluated PFTs, respiratory symptoms, inhaled bronchodilator medication use, and hematological variables to determine the effectiveness of *Z. multiflora* in patients with COPD. Three groups of patients were randomly assigned: two groups received varying dosages of *Z. multiflora* extract, and the third group received a placebo. The findings showed that after receiving *Z. multiflora* treatment, FEV_1_ values significantly improved. When compared to baseline values, respiratory symptoms significantly decreased after 1 and 2 months of extract administration. By the end of the study, the use of inhaled bronchodilator medications in the extract-receiving groups had considerably reduced. Additionally, 1-2 months following extract therapy, a decrease in total white blood cell counts relative to baseline values was noted. After two months of treatment, compared to month one, neutrophil levels significantly dropped in the groups who received *Z. multiflora* extract (29). Various experimental studies evaluating the effect of *Z. multiflora* extract on animal models of COPD are in line with the results of the above clinical findings (170, 171).

### Effect of Z. multiflora constituents

*Carvacrol*

The effect of carvacrol on wheezing, FEV_1_, and plasma nitrite levels in asthmatic patients was the goal of an investigation. Two groups of asthmatic patients were assigned: one for carvacrol and the other for a placebo. FEV_1_, nitrite levels, and wheezing during the day and exercise were measured three times. Comparing the carvacrol-treated groups to the baseline, the results showed a significant decrease in daytime and exercise-related wheeze. Additionally, after therapy, nitrite levels dramatically dropped in the carvacrol groups, and FEV_1_ greatly increased in these groups. On the other hand, during the course of the study, the examined parameters showed very little change in the placebo group (155). The effects of carvacrol on the animal model of asthma were indicated in experimental studies, which supported the above clinical findings (167, 172-174).

The effects of carvacrol on PFTs, hematological indices, and oxidant/anti-oxidant biomarkers in individuals with lung disorders caused by exposure to sulfur mustard gas 27–30 years ago were investigated. Patients were divided into two groups: one received carvacrol, and the other received a placebo. The findings showed that, after two months, PEF in the carvacrol-treated group was considerably higher than baseline. Additionally, following months one and two, the levels of thiol, SOD, and CAT increased substantially in the carvacrol-treated group, whereas the total white blood cell count, neutrophil count, and MDA levels significantly dropped (175).

Important results were found in a study assessing the effects of carvacrol on patients exposed to sulfur mustard. Serum levels of ILs and IFN-γ changed significantly after receiving carvacrol for two months. In particular, IL-10 and IFN-γ levels rose in month two, but IL-2, IL-4, IL-6, and IL-8 levels significantly decreased in months one and two when compared to baseline. In month two, the IFN-γ/IL-4 ratio also improved. Furthermore, in comparison to the baseline, the PFTs of the carvacrol-treated group, which included MMEF and maximum expiratory flow at 25, 50, and 75% of vital capacity (MEF_25_, MEF_50_, and MEF_75_), showed notable improvements in months one and two (176). 

Likewise, patients with a history of sulfur mustard exposure lasting more than 20 years were assigned to either a placebo or a carvacrol-treated group in a research study to examine the effects of carvacrol on inflammatory markers and respiratory symptoms. At baseline and one and two months after the start of treatment, serum levels of TNF-α, MCP-1, VEGF, EGF, FEV_1_, and respiratory symptoms such as chest wheeze, night wheeze, night cough, and cough and wheeze during exercise were measured. The results showed that the FEV_1_ of the carvacrol group increased significantly at month two when compared to baseline, and that this improvement continued from month one to month two. In comparison to baseline, respiratory symptoms, chest wheeze, and night wheeze greatly decreased in months 1 and 2, but only in month 2 did night cough and cough and wheeze during exercise significantly decrease. Additionally, TNF-α, EGF, and VEGF levels of the carvacrol group dropped at months 1 and 2 in comparison to baseline, whereas MCP-1 levels only markedly declined at month 2 in comparison to baseline (177). 

Several clinical trials have examined the effects of *Z. multiflora* and carvacrol on a variety of respiratory disorders, with promising findings. *Z. multiflora* improved PFTs, decreased respiratory symptoms, and altered inflammatory markers and oxidative stress levels, most likely due to its anti-inflammatory, anti-oxidant, and immunomodulatory properties. While these studies developed useful information, they had limitations such as small sample sizes and short durations. Future plans include conducting larger, multicenter trials with more extended follow-up periods to evaluate efficacy and safety, investigating optimal doses and formulations, assessing interactions with existing therapies, and exploring the exact biochemical pathways involved in greater detail. The consistent findings highlight the potential of *Z. multiflora* and carvacrol as therapeutic agents for respiratory diseases, suggesting the need for additional research in this area.

## Possible mechanisms of action

Dyspnea is influenced by various physiological processes, such as oxidative stress, inflammation, and immune dysregulation. Herbal treatments have emerged as promising strategies for treating dyspnea by targeting these major pathways.

**Figure 3:**
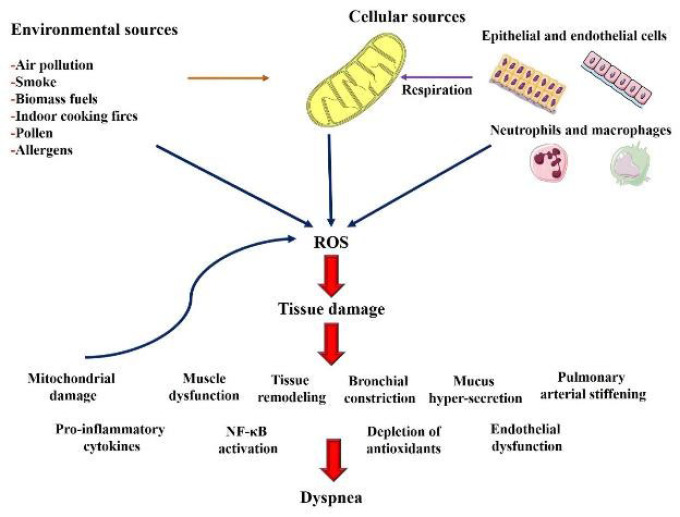
Impact of oxidative stress on dyspnea induction (Images from https://smart.servier.com and https://www.freepik.com)

**Figure 4:**
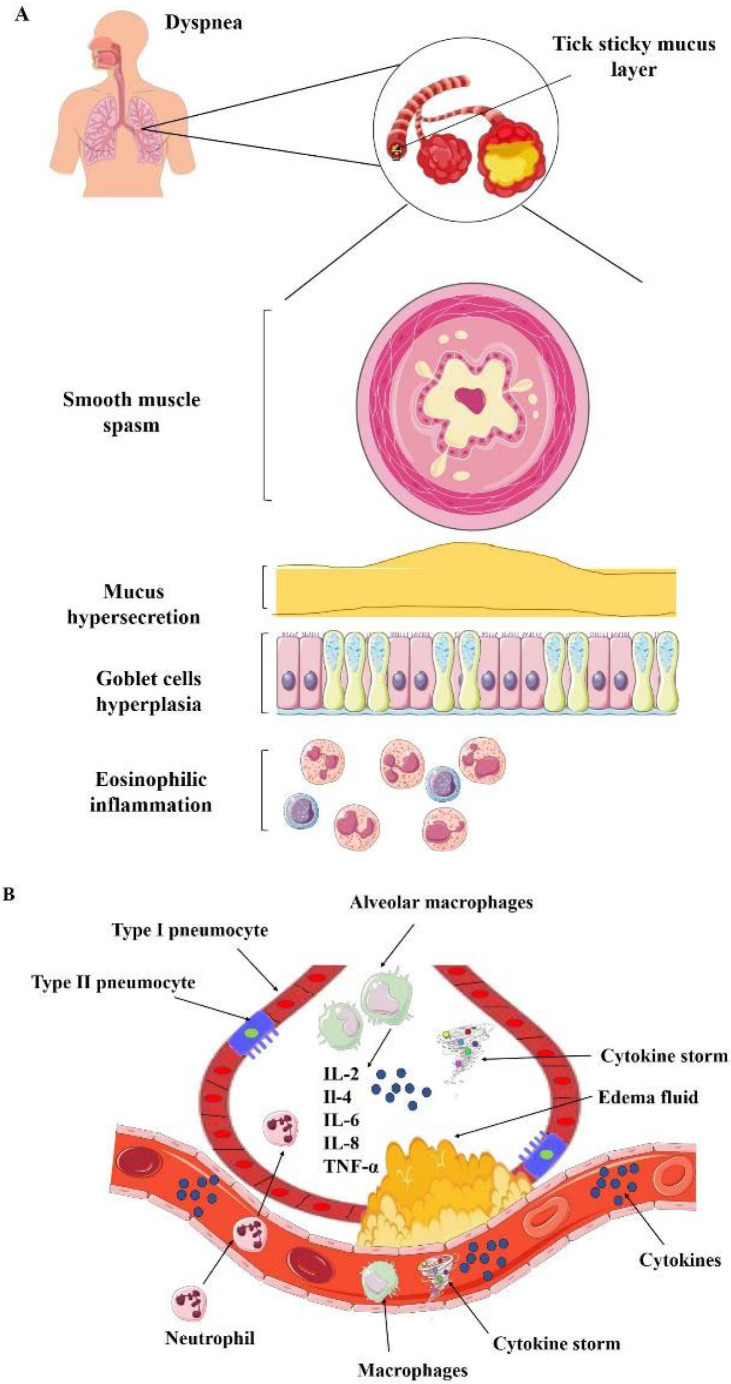
Impact of inflammation and immune dysregulation on dyspnea induction in A: Respiratory tract and B: Alveolus and pulmonary capillaries (Images from https://smart.servier.com and https://www.freepik.com)

1. Oxidative stress and dyspnea: Oxidative stress, or an imbalance between ROS generation and anti-oxidant defenses, is a major cause of respiratory distress (178). In disorders such as COPD and asthma, increased oxidative stress causes airway inflammation and decreases lung function, worsening dyspnea (179) (Figure 3). The herbs reported in this review are high in anti-oxidants, which scavenge ROS, reduce oxidative damage, and protect respiratory tissues, thereby reducing dyspnea symptoms.

2. Inflammation and dyspnea: Chronic inflammation in the airways is a hallmark of respiratory disorders and a major cause of dyspnea. Inflammatory mediators trigger airway narrowing, mucus hypersecretion, and tissue remodeling, all of which can worsen breathing problems (180) (Figure 4). These herbs possess strong anti-inflammatory properties, which inhibit pro-inflammatory cytokines and enzymes, thereby reducing airway inflammation and improving respiratory function in dyspneic situations.

3. Immuno-dysregulation and dyspnea: Abnormal immunological responses can lead to chronic inflammation in the respiratory system, exacerbating dyspnea. Immunomodulation is crucial for restoring immunological balance and preventing excessive inflammation, which can lead to respiratory distress. These herbs have immunomodulatory effects that regulate immunological responses, modify inflammatory pathways, and reduce airway hyper-responsiveness, providing relief for dyspnea (181, 182).

Finally, the anti-oxidant, anti-inflammatory, and immunomodulatory effects of herbal treatments present a comprehensive and integrative approach to managing dyspnea. By addressing the underlying mechanisms associated with respiratory distress, these herbs offer a promising opportunity for improving respiratory health outcomes and enhancing the quality of life for individuals suffering from breathing difficulties. 

## Future perspectives

In the future, the field of herbal treatments for respiratory disorders, which address dyspnea, presents a complex web of possibilities and challenges that require investigation and clarification. The current level of research reveals several important future perspectives:

1. The development of standardized procedures for the application of herbal remedies in the treatment of dyspnea is an essential area for progress. To guarantee safety and repeatability throughout research and clinical practice, precise recommendations on doses, formulations, and quality control procedures are crucial.

2. Exploring the fundamental mechanisms of herbal therapies can provide novel opportunities for treating dyspnea and respiratory symptoms. By identifying the molecular targets and processes involved, researchers may establish a path for the development of targeted and effective treatments.

3. Longitudinal studies that focus on the long-term safety and efficacy of herbal therapies are critical for determining their long-term benefits and potential side effects. This research can shed light on the long-term efficacy of treatments and help clinicians make better decisions.

4. Investigating the synergistic effects of herbal therapies in conjunction with conventional treatments shows promise for increasing therapeutic outcomes and symptom management. Investigating the best combinations and dose regimes can lead to individualized and effective treatment strategies.

5. Future research should focus on patient-centered outcomes and preferences to tailor interventions to individual needs and experiences. Applying a patient-centered approach can improve treatment adherence, satisfaction, and overall quality of care.

6. Collaborations among academics, physicians, herbalists, and other healthcare professionals can promote a comprehensive and integrated approach to dyspnea management. Interdisciplinary efforts may encourage innovation and improve patient outcomes by bringing together various knowledge and viewpoints.

By following these future perspectives and doing research in these areas, the field of herbal treatments for dyspnea in respiratory disorders has the potential to revolutionize respiratory care. The future of herbal therapies in conventional respiratory healthcare practices appears to be bright, owing to thorough scientific study, teamwork, and a consistent commitment to improving patient well-being.

## Limitations and future directions

### Limitations of the evidence

While this review synthesizes findings on herbal interventions for dyspnea, several limitations should be acknowledged. The included studies vary in design, sample size, dosage, and treatment duration, making direct comparisons challenging. Additionally, some studies lack standardized methodologies, which may affect the consistency of reported outcomes. Further high-quality clinical trials with well-defined protocols are needed to validate the efficacy and safety of herbal therapies.

### Limitations of the review process

This review was conducted as a narrative synthesis, relying on published peer-reviewed studies rather than a systematic assessment with formal risk of bias evaluations. The selection of articles was performed manually by a single reviewer, which, while structured, may introduce selection bias. Additionally, non-English studies were excluded, which could limit perspectives from different research communities.

## Implications for practice, policy, and future research

Herbal medicine presents a promising complementary approach for dyspnea management, offering potential bronchodilatory, anti-inflammatory, and anti-oxidant effects. However, to facilitate clinical integration, standardized formulations, dosage optimization, and long-term safety evaluations are necessary. Future research should focus on large-scale, randomized controlled trials that explore synergistic effects with conventional treatments and address potential interactions. From a policy perspective, further regulatory frameworks are required to ensure quality control, accessibility, and safety in herbal therapeutics.

## Conclusion

**Figure 5:**
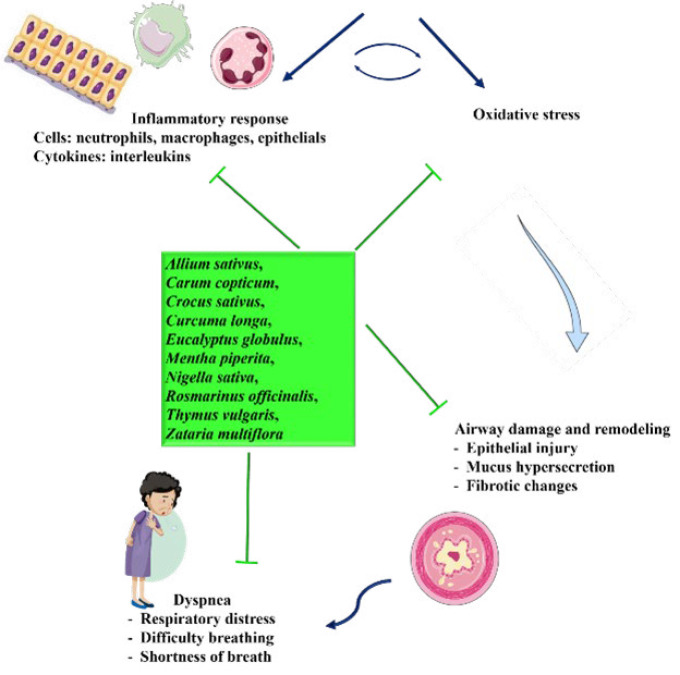
Therapeutic effects of herbal medicines on dyspnea (Images from https://smart.servier.com and https://www.freepik.com)

Based on the narrative review on herbal remedies for alleviating dyspnea in respiratory conditions, it is evident that various herbal interventions show promising potential in managing dyspnea and improving respiratory health outcomes (Figure 5). Research on *A. sativus* supplements, *C. copticum*, *C. sativus*, crocin, curcumin, *E. globulus*, *M. piperita* essential oil, L-menthol, *N. sativa*, *R. officinalis* extract, 1,8-cineol, *T. vulgaris*, *Z. multiflora*, and carvacrol has provided valuable insights into their efficacy in addressing dyspnea and related respiratory symptoms. These herbal remedies exhibit diverse mechanisms of action, including bronchodilatory, anti-inflammatory, anti-oxidant, and immunomodulatory properties that contribute to their therapeutic effects. 

While the trials show promising effects, limitations such as different dosages, sample sizes, and treatment durations prevent direct comparisons and generalizability. Future research should focus on larger randomized controlled trials with standardized methodologies, investigating optimal dosages, long-term safety profiles, and potential interactions with conventional treatments in order to improve understanding and application of herbal remedies in respiratory care.

Addressing these research gaps and investigating the synergistic effects of herbal interventions with existing therapies presents a significant opportunity to optimize dyspnea management strategies, improve respiratory health outcomes, and provide personalized care for people with respiratory problems. The findings highlight the potential of herbal remedies as helpful supplemental therapies in the management of dyspnea and respiratory disorders.

**Table 1:** Effect of *Allium sativum*, *Carum copticum*, and *Crocus sativus *on dyspnea

Compound	Study design	Doses/Duration	Results	Ref.
*A. sativum* powder	15 participants with HPS	6 months, PO	↑ PO_2_ ↓ Alveolar-arterial gradient, exertional dyspnea	(35)
Dried *A. sativum*	15 children with HPS	1 g/1.73 m^2^, 5 months, PO	↑ Mean arterial oxygen pressure, PaO_2_	(36)
*A. sativum* capsules (Gallecina)	72 non-critically ill COVID-19 patients	90 mg, three times a day, 5 days, PO	↓ Body temperature, supplementary oxygen	(37)
*A. sativum*	20 Healthy overweight adult	3-4 g, PO	↑ FEF25-75% ↓ FVC, FEV_1_	(38)
*A. sativum* powder	22 patients with pulmonary tuberculosis	5 g, three times a day, 7 days, PO	↓ Pulmonary tuberculosis signs and symptoms	(39)
*C. copticum* boiled extract	15 asthmatic patients	0.125, 0.25 ml/kg of 10 g% boiled extract, PO	↑ PFTs values	(45)
Dried *C. sativus* stigma	80 individuals with mild/moderate allergic asthma	50 mg, twice a day, 8 weeks, PO	↑ FEF, FVC, FEV_1_, FEV_1_/FVC ratio ↓ hs-CRP, anti-HSP70 concentrations	(59)
Dried *C. sativus* stigma	80 samples with mild/moderate persistent allergic asthma	100 mg, 8 weeks, PO	↑ Sleep quality, activity levels ↓ Use of salbutamol spray, frequency of shortness of breath, severity of asthma	(60)
Crocin	23 COPD patients	30 mg, 12 weeks, PO	↑ Total antioxidant capacity, 6MWD ↓ Blood NF-κB levels, total oxidant status	(62)
Crocin	57 participants with COPD	15 mg, twice a day, 12 weeks, PO	↑ IL-6 levels, 6MWD, FEV_1_, FEV_1_/FVC ↓ Serum TNF-α levels	(63)

6MWD: 6-Minute walk distance; COPD: Chronic obstructive pulmonary disease; COVID-19: Coronavirus disease 2019; FEF: Forced expiratory flow; FEV1: Forced expiratory volume in 1 second; FVC: Forced vital capacity; HPS: Hepatopulmonary syndrome; hs-CRP: High-sensitivity C-reactive protein; HSP70: Heat shock protein 70; IL-6: Interleukin-6; NF-κB: Nuclear factor Kappa B; PO.: Per Os (by mouth); PaO_2_: Partial pressure of oxygen in arterial blood; PFTs: Pulmonary function tests; PO_2_: Partial pressure of oxygen; TNF-α: Tumor necrosis factor-alpha

**Table 2:** Effect of *Curcuma longa *and *Eucalyptus globulus* on dyspnea

Compound	Study design	Doses/Duration	Results	Ref.
Curcumin	60 mild/moderate bronchial asthma	500 mg, 30 days, PO	↑ Mean FEV_1_ values ↓ Airway obstruction	(74)
Curcumin	40 asthmatic participants	750 mg, twice daily, two months, PO	↑ FEV_1_ and FEV_1_/FVC ratio ↓ Neutrophil count	(76)
Curcumin	23 patients with chronic bronchial asthma	750 mg, twice a day, 2 weeks, PO	↑ FEV_1_, predicted FVC, FEV_1_/FVC ratio, PEF values ↓ IL-6 levels	(77)
Curcuminoids	Male patients with chronic SM-induced pulmonary complications	500 mg, TID, weeks, PO	↑ FEV_1_/FVC ratio - FEV_1_ and FVC stayed similar between groups	(78)
Nanocurcumin	40 individuals with COVID-19	160 mg, 14 days, PO	↓ IL-1β and IL-6 gene expression and secretion levels, fever, cough, dyspnea	(80)
Nanocurcumin	60 mild/moderate COVID-19 participants	40 mg, twice a day, 2 weeks, PO	↑ Lymphocyte counts ↓ Respiratory symptoms (e.g., dyspnea)	(81)
Nanocurcumin	53 moderately obese menopause women	40 mg capsules, twice a day, twice a week, 8 weeks, PO	↑ Oxygen pulse and VO_2_max by both methods ↑ Muscular fitness↑ FVC ↑ Physical performance and cardiorespiratory fitness	(82)
Curcumin	4551 adults	Curcumin-rich curry consumption	- Non-linear correlations with variables like COPD prevalence and FEV_1_/height^2^	(83)
*E. globulus* essential oil	15 individuals with post-COVID syndrome	Nebulized three drops, twice/day, 4 weeks	↓ Anxiety, back pain, dyspnea	(89)
Nebulized* E. globulus* solution	70 intubated patients	4 ml (5%) 3 times a day since intubation, 3 days	↑ PaO_2_, SaO_2_ ↓ PIP	(90)
Cineole	242 stable COPD patients	200 mg, PO, 3 times/day, 6 months	↑ PFTs, quality of life ↓Severity, duration, and frequency of dyspnea	(91)
Cineole	247 asthmatic patients	200 mg, PO, 3 times/day, 6 months,	↑PFTs, quality of life ↓ Asthma symptoms, dyspnea	(92)

COPD: Chronic obstructive pulmonary disease; COVID-19: Coronavirus disease 2019; CRP: C-reactive protein; PFTs: Pulmonary function tests; FEV1: Forced expiratory volume in 1 second; FVC: Forced vital capacity; IL: Interleukin; PO: Per Os (by mouth); PaO_2_: Partial pressure of oxygen in arterial blood; PIP: Peak inspiratory pressure; SaO_2_: Arterial oxygen saturation; VO_2_: Oxygen consumption

**Table 3:** Effect of *Mentha piperita* on dyspnea

Compound	Study design	Doses/Duration	Results	Ref.
*M. piperita* essential oil	12 healthy male students	0.05 ml, 10 days, PO	↑FVC, PEF, PIF, exhaustion, VO_2_, VCO_2_	(100)
L-menthol	11 normal participants	300 mg in 250 ml total respiratory space	↓ Respiratory discomfort, VAS values	(102)
L-menthol	64 patients	3 ml 1% l-menthol solution	↑ PEF ↓ Coughing and dyspnea	(103)
L-menthol	28 COPD patients	-	↓ Air hunger, anxiety, breathing discomfort, fear, and breathing effort	(104)
L-menthol	Chronic breathlessness syndrome	Circular patch with a diameter of 15 mm	↓ Dyspnea on exertion	(105)
L-menthol	13 male runners	0.01% solution, 25 ml	↑ Running time, breathing comfort, dyspnea threshold, endurance capacity	(106)

COPD: Chronic obstructive pulmonary disease; FVC: Forced vital capacity; PO.: Per Os (by mouth); PaO_2_: Partial pressure of oxygen in arterial blood; PEF: Peak expiratory flow; PIF: Peak inspiratory flow; VAS: Visual analog scale; VCO_2_: Carbon dioxide production; VO_2_: Oxygen consumption

**Table 4:** Effect of *Nigella sativa*, Rosmarinus* officinalis*, and *Thymus vulgaris* on dyspnea

Compound	Study design	Doses/Duration	Results	Ref.
*N. sativa* SBAE	29 asthmatic patients	15 ml/kg of 0.1 g%, PO boiled extract, 3 months,	↑PFTs, FVC, MMEF	(114)
*N. sativa* SBAE	15 asthmatic patients	50, 100 mg/kg, PO	↑ PFTs ↓ Extract effects on PFTs compared to theophylline	(115)
Boiled* N. sativa* seed	18 chronic asthmatic patients	100 mg/kg, 21 days, inhalation	↑ FEV_1_%, FVC ↓ Asthma attacks	(116)
*N. sativa* oil	60 Asthmatic patients	500 mg, twice daily, 4 weeks, PO	↑ ACT score, FEV_1_ ↓ Blood eosinophils	(117)
*N. sativa* oil	76 patients with partially controlled asthma	1, 2 g/day, 3 months, PO	↑ FEV_1_ and FEF 25-75%, PEF, 6 and 12 weeks	(118)
*N. sativa* SBAE	40 chemical war victims	0.375 ml/kg/bw, PO (50 mg/ml extract), 2 months	↑PFTs, FVC, FEV_1_ ↓Wheezing, coughing	(119)
*N. sativa* oil	91 COPD patients	1 g, twice daily, 3 months, PO	↑CAT, GPx, GSH, SOD, vitamin C, and E, PFTs ↓IL-6, TBARS, TNF-α	(120)
*N. sativa* plus vitamin D3	120 COVID-19 patients, mild/moderate	*N. sativa*: 900 mg vitamin D3: 2,000 IU, 2 weeks, PO	↓ symptoms (cough, dyspnea, fatigue)	(121)
*R. officinalis* leaves extract	44 asthmatic patients resistant to routine treatments	50 ml (each one ml contained 200 mg herbal extract), three times a day, for a month, PO	↑ ACT scores ↓ Coughing, sputum production, wheezing, exhaled air nitric oxide	(137)
*R. officinalis* hydroalcoholic extract	77 COPD patients	500 mg, twice a day, 2 months, PO	↑ gains in IADL after controlling for OSA	(138)
1,8-cineol	32 steroid-dependent asthmatic patients	200 mg, three times a day, 12 weeks, PO	↓ Daily prednisolone dose Absence of ↓ prednisone on PFTs	(139)
*T. vulgaris* powder	60 mild to moderate asthma-exacerbated child	20 mg/kg, three times a day, for a week, PO	↑ FEV_1_ ↓Activity-induced cough	(146)
*T. vulgaris* oil	COVID-19 patients	Three times a day, 5 days, inhalation	↑ SPO_2_ ↓Cough, shortness of breath, respiratory rate	(147)

ACT: Asthma control test; CAT: Catalase; COPD: Chronic obstructive pulmonary disease; COVID-19: Coronavirus disease 2019; FEF: Forced expiratory flow; FEV1: Forced expiratory volume in 1 second; FVC: Forced vital capacity; GPx: Glutathione peroxidase; GSH: Glutathione; IADL: Instrumental activities of daily living; IL-6: Interleukin-6; MMEF: Maximal mid-expiratory flow; OSA: Obstructive sleep apnea; PO: Per Os (by mouth); PCR: Polymerase chain reaction; PEF: Peak expiratory flow; PEFR: peak expiratory flow rate; PFTs: Pulmonary function tests; SBAE: Seed boiled aqueous extract; SOD: Superoxide dismutase; SPO_2_: Peripheral oxygen saturation; TBARS: Thiobarbituric acid reactive substances; TNF-α: Tumor necrosis factor-alpha

**Table 5:** Effect of *Z. multiflora* on dyspnea

Compound	Study design	Doses/Duration	Results	Ref.
Z*. multiflora* extract	40 asthmatic patients	5, 10 mg/kg, 2 months, PO	↑ FEV_1_% ↓Wheezing, nitrite levels	(155)
Z*. multiflora* extract	36 asthmatic patients	5, 10 mg/kg, 2 months, PO	↑ PFTs ↓Clinical symptoms, oxidative stress	(156)
*Z. multiflora* extract	18 asthmatic patients	20 mg/kg, PO	↑PFT values between 30 and 180 min	(153)
Z*. multiflora* extract	47 veterans exposed to SM	5, 10 mg/kg, 2 months, PO	↑ PEF, serum thiol, SOD, CAT, FVC ↓ MDA, WBC	(160)
Z*. multiflora* extract	34 veterans exposed to SM	5, 10 mg/kg, 2 months, PO	↑ FEV_1_ values ↓ Cough, chest wheezing, EGF, MCP-1, TNF-α, VEGF	(161)
Z*. multiflora* extract	34 veterans exposed to SM	5, 10 mg/kg, 2 months, PO	↑ IL-10, IFN-γ, MMEF, MEF25, 50, 75 ↓Serum levels of IL-2, IL-6, IL-8	(162)
Z*. multiflora* extract	45 patients with COPD	3, 6 mg/kg, 2 months, PO	↑ FEV_1_, thiol, SOD, and CAT ↓ Sputum, chest wheezing, dyspnea, MDA, nitrite, CRP levels	(163)
Z*. multiflora* extract	41 patients with COPD	3, 6 mg/kg, 2 months, PO	↑ FVC, FEV_1_, PEF ↓Serum levels of TNF-α and IL-8, cough, chest tightness, dyspnea	(169)
Z*. multiflora* extract	45 patients with COPD	3, 6 mg/kg, 2 months, PO	↑ FEV_1_ values ↓Respiratory symptoms, inhaled bronchodilator use, and WBC counts	(29)
Carvacrol	40 asthmatic patients	1.2 mg/kg, 2 months, PO	↑ FEV_1_% ↓ Wheezing, nitrite levels	(155)
Carvacrol	20 patients exposed to SM	3.6 mg/kg, 2 months, PO	↑ PEF, serum levels of thiol, SOD, CAT ↓ WBC, MDA level	(175)
Carvacrol	20 patients exposed to SM	1.2 mg/kg, three times a day, 2 months, PO	↑ IL-10 and IFN‐γ levels, IFN-γ/IL-4 ratio, MMEF, MEF25, MEF50, MEF75 ↓ IL-2, IL-4, IL-6, IL-8 levels	(176)
Carvacrol	21 patients exposed to SM	1.2 mg/kg, three times a day, 2 months, PO	↑ FEV_1_ ↓ Chest wheeze, cough and wheeze, TNF-α, EGF, VEGF, and MCP-1 levels	(177)
Carvacrol	30, 40 healthy subjects	2 mg/kg, a month, PO	↑ FEV_1_	(183, 184)

CAT: Catalase; COPD: Chronic obstructive pulmonary disease; CRP: C-reactive protein; EGF: Epidermal growth factor; FEV1: Forced expiratory volume in one second; FVC: Forced vital capacity; IFN-γ: Interferon-gamma; IL: Interleukin; MCP-1: Monocyte chemotactic protein 1; MDA: Malondialdehyde; MEF: Maximal expiratory flow; MMEF: Maximal mid expiratory flow; PO: Per Os (by mouth); PEF: Peak expiratory flow; PFTs: Pulmonary function tests; SM: Sulfur mustard; SOD: Superoxide dismutase; TNF-α: Tumor necrosis factor-alpha; VEGF: Vascular endothelial growth factor

## Data Availability

No new data were created or analyzed during this study. Data sharing does not apply to this article.

## Declaration

During the preparation of this work, the author(s) used ChatGPT and Quillbot to rephrase to reduce plagiarism and improve the language and grammar. After using this tool/service, the author(s) reviewed and edited the content as needed and take(s) full responsibility for the content of the publication.

## References

[1] Coccia CB, Palkowski GH, Ntusi N, Schweitzer B, Motsohi T (2016). Dyspnoea: Pathophysiology and a clinical approach. S Afr Med J.

[2] Gruenberger JB, Vietri J, Keininger DL, Mahler DA (2017). Greater dyspnea is associated with lower health-related quality of life among European patients with COPD. Int J Chron Obstruct Pulmon Dis.

[3] Zhang J, Zhang Y, Yin Y, Feng Y, Zhang R, Meng H (2024). ‘Fear, uncertain, tired psychological distress among pulmonary hypertension patients: a qualitative interview study. BMC psychiatry.

[4] Ballasingam A, Islahudin F, Abdul-Aziz S-A, Makmor-Bakry M (2023). Effect of dyspnea, quality of life, and well-being in postacute COVID-19 patients: A prospective cohort study. Asian Journal of Pharmaceutical Research and Health Care.

[5] Fukushi I, Pokorski M, Okada Y (2021). Mechanisms underlying the sensation of dyspnea. Respir Investig.

[6] Rawashdeh AI (2024). The significance of FEV1 and FEV1/FVC in COPD diagnosis. Jordan J Biol.

[7] Stanojevic S, Kaminsky DA, Miller MR, Thompson B, Aliverti A, Barjaktarevic I (2022). ERS/ATS technical standard on interpretive strategies for routine lung function tests. Eur Respir J.

[8] Stickland MK, Neder JA, Guenette JA, O’Donnell DE, Jensen D (2022). Using cardiopulmonary exercise testing to understand dyspnea and exercise intolerance in respiratory disease. Chest.

[9] Hosseini M, Pkan P, Rakhshandeh H, Aghaie A, Sadeghnia HR, Rahbardar MG (2011). The effect of hydro-alcoholic extract of citrus flower on pentylenetetrazole and maximal electroshock-induced seizures in mice. World Appl Sci J.

[10] Emadi SA, Ghasemzadeh Rahbardar M, Mehri S, Hosseinzadeh H (2022). A review of therapeutic potentials of milk thistle (Silybum marianum L ) and its main constituent, silymarin, on cancer, and their related patents. Iran J Basic Med Sci.

[11] Ardakanian A, Ghasemzadeh Rahbardar M, Omidkhoda F, Razavi BM, Hosseinzadeh H (2022). Effect of alpha-mangostin on olanzapine-induced metabolic disorders in rats. Iran J Basic Med Sci.

[12] Jalali J, Ghasemzadeh Rahbardar M (2023). Ameliorative effects of Portulaca oleracea L (purslane) and its active constituents on nervous system disorders: A review. Iran J Basic Med Sci.

[13] Omidvar Tehrani S, Ghasemzadeh Rahbardar M, Shoorgashti K, Dehghan Nayeri MJ, Mohammadpour AH, Hosseinzadeh H (2023). Evaluation of berberine pellet effect on clinical recovery time in COVID-19 outpatients: A pilot clinical trial. Avicenna J Phytomed.

[14] Boskabady MH, Ghasemzadeh Rahbardar M, Nemati H, Esmaeilzadeh M (2010). Inhibitory effect of Crocus sativus (saffron) on histamine (H1) receptors of guinea pig tracheal chains. Pharmazie.

[15] Boskabady MH, Rahbardar MG, Jafari Z (2011). The effect of safranal on histamine (H1) receptors of guinea pig tracheal chains. Fitoterapia.

[16] Boskabady MH, Jafari Z, Pouraboli I, Babazade B, Rahbardar MG (2012). Anti-cholinergic effect of Zataria multiflora Boiss on guinea pig tracheal chains. Nat Prod Res.

[17] Popova A, Mihaylova D, Spasov A (2021). Plant-based remedies with reference to respiratory diseases – A review. The Open Biotechnology Journal.

[18] Ghaleb Dailah H (2022). The ethnomedicinal evidences pertaining to traditional medicinal herbs used in the treatment of respiratory illnesses and disorders in Saudi Arabia: A review. Saudi J Biol Sci.

[19] Azman S, Sekar M, Bonam SR, Gan SH, Wahidin S, Lum PT (2021). Traditional medicinal plants conferring protection against ovalbumin-induced asthma in experimental animals: A review. J Asthma Allergy.

[20] Rahman MM, Bibi S, Rahaman MS, Rahman F, Islam F, Khan MS (2022). Natural therapeutics and nutraceuticals for lung diseases: Traditional significance, phytochemistry, and pharmacology. Biomed Pharmacother.

[21] Avicenna The Canon of Medicine.

[22] Khorasani MHA Makhzan al-Adwiya.

[23] Momen Tonekaboni SM Tohfat al-Momenin.

[24] Nath V, Buragohain P, Sharma H (2021). A review on some medicinal plants of Northeast India used in the treatment of respiratory disorders. Curr Trends Pharm Res.

[25] Shakeri F, Amin F, Marefati N, Roshan NM, Boskabady M, Boskabady MH (2023). Effect of Allium cepa extract on total and differential WBC, TP level, oxidant and antioxidant biomarkers, and lung pathology in ovalbumin-sensitized rats. Allergol Immunopathol.

[26] Ghobadi H, Aslani F, Boskabady MH, Saadat S, Aslani MR (2024). Saffron (Crocus sativus) and its constituents in ovalbumin-induced asthma model: a preclinical systematic review and meta-analysis. Front Pharmacol.

[27] Aminian AR, Mohebbati R, Boskabady MH (2021). The effect of Ocimum basilicum L and its main ingredients on respiratory disorders: An experimental, preclinical, and clinical review. Front Pharmacol.

[28] Khazdair MR, Saadat S, Aslani MR, Shakeri F, Boskabady MH (2021). Experimental and clinical studies on the effects of Portulaca oleracea L and its constituents on respiratory, allergic and immunologic disorders, a review. Phytother Res.

[29] Ghorani V, Rajabi O, Mirsadraee M, Amini M, Ghaffari S, Boskabady MH (2024). Zataria multiflora affects pulmonary function tests, respiratory symptoms, bronchodilator drugs use and hematological parameters in chronic obstructive pulmonary disease patients: A randomized doubled-blind clinical trial. J Ethnopharmacol.

[30] Tudu CK, Dutta T, Ghorai M, Biswas P, Samanta D, Oleksak P (2022). Traditional uses, phytochemistry, pharmacology and toxicology of garlic (Allium sativum), a storehouse of diverse phytochemicals: A review of research from the last decade focusing on health and nutritional implications. Front Nutr.

[31] Subroto E, Cahyana Y, Tensiska M, Lembong F, Filianty E, Kurniati E (2021). Bioactive compounds in garlic (Allium sativum L) as a source of antioxidants and its potential to improve the immune system: a review. Food Res.

[32] Savira M, Sari DK, Machrina Y, Widjaja SS, Unitly AJA, Ilyas S (2023). Anti inflammatory action of Allium sativum ethanol extract to prevent lung damage in smoker rat model. Med Arch.

[33] Okunye OL, Idowu PA, Adeleke EO, Babalola CO (2020). Antimicrobial activity of garlic (Allium sativum) on selected uropathogens from cases of urinary tract infection. Ann Trop Pathol.

[34] Patiño-Morales CC, Jaime-Cruz R, Sánchez-Gómez C, Corona JC, Hernández-Cruz EY, Kalinova-Jelezova I (2021). Antitumor effects of natural compounds derived from Allium sativum on neuroblastoma: an overview. Antioxidants.

[35] Abrams GA, Fallon MB (1998). Treatment of hepatopulmonary syndrome with Allium sativum L (garlic): A pilot trial. J Clin Gastroenterol.

[36] Najafi Sani M, Kianifar HR, Kianee A, Khatami G (2006). Effect of oral garlic on arterial oxygen pressure in children with hepatopulmonary syndrome. World J Gastroenterol.

[37] Taghavi MR, Tavanaei Tamanaei T, Oghazian MB, Tavana E, Mollazadeh S, Niloofar P (2023). Effectiveness of fortified garlic extract oral capsules as adjuvant therapy in hospitalized patients with Coronavirus Disease 2019: A triple-blind randomized controlled clinical trial. Curr Ther Res Clin Exp.

[38] Azekhumen G, Okolie L (2023). Estimation of the combined effect of garlic and moderate exercise on lung function in healthy overweight adult human subjects with no history of respiratory defects. J Appl Sci Environ Manage.

[39] Retno RT, Lajuward AI (2024). Effect of garlic powder (Allium sativum Linn) on reducing the sign and symptoms of pulmonary tuberculosis. J Health Sci.

[40] Boskabady MH, Alitaneh S, Alavinezhad A (2014). Carum copticum L. : a herbal medicine with various pharmacological effects. Biomed Res Int.

[41] Izadi M, Jorf SAM, Nikkhah M, Moradi S (2021). Antifungal activity of hydrocolloid nano encapsulated Carum copticum essential oil and Peganum harmala extract on the pathogenic fungi Alternaria alternata. Physiol Mol Plant Pathol.

[42] Alavinezhad A, Boskabady MH (2014). Antiinflammatory, antioxidant, and immunological effects of Carum copticum L and some of its constituents. Phytother Res.

[43] Boskabady MH, Alizadeh M, Jahanbin B (2007). Bronchodilatory effect of Carum copticum in airways of asthmatic patients. Therapie.

[44] Mohammadi M, Masoumipour F, Hassanshahian M, Jafarinasab T (2019). Study the antibacterial and antibiofilm activity of Carum copticum against antibiotic-resistant bacteria in planktonic and biofilm forms. Microb Pathog.

[45] Boskabady MH, Alizadeh M, Jahanbin B (2007). Bronchodilatory effect of Carum copticum in airways of asthmatic patients. Therapie.

[46] Ghasemzadeh Rahbardar M, Hosseinzadeh H (2023). A review of how the saffron (Crocus sativus) petal and its main constituents interact with the Nrf2 and NF-κB signaling pathways. Naunyn Schmiedebergs Arch Pharmacol.

[47] Zolfaghari Farajerdi M, Rajabian F, Razavi BM, Ghasemzadeh Rahbardar M, Khajavi Rad A, Amoueian S (2024). Evaluating the effect of crocin on contrast-induced nephropathy in rats. Avicenna J Phytomed.

[48] Rajabalizadeh R, Ghasemzadeh Rahbardar M, Razavi BM, Hosseinzadeh H (2024). Renoprotective effects of crocin against colistin-induced nephrotoxicity in a rat model. Iran J Basic Med Sci.

[49] Aminifard T, Mehri S, Ghasemzadeh Rahbardar M, Rajabian F, Khajavi Rad A, Hosseinzadeh H (2024). Trans-sodium crocetinate suppresses apoptotic and oxidative response following myoglobin-induced cytotoxicity in HEK-293 cells. Iran J Basic Med Sci.

[50] Boskabady MH, Farkhondeh T (2016). Antiinflammatory, antioxidant, and immunomodulatory effects of Crocus sativus L and its main constituents. Phytother Res.

[51] Boskabady MH, Mokhtari-Zaer A, Khazdair MR, Memarzia A, Gholamnezhad Z, Crocus sativus L (2020). (Saffron) and its components relaxant effect on smooth muscles and clinical applications of this effect. Saffron.

[52] Ghasemzadeh Rahbardar M, Shakeri F, Boskabady MH (2025). Medicinal plants and histamine (H1) receptors: An updated review. Pharm Sci.

[53] Naraki K, Ghasemzadeh Rahbardar M, Razavi BM, Aminifar T, Khajavi Rad A, Amoueian S (2024). The power of trans-sodium crocetinate: exploring its renoprotective effects in a rat model of colistin-induced nephrotoxicity. Naunyn Schmiedebergs Arch Pharmacol.

[54] Rajabian F, Mehri S, Razavi BM, Khajavi Rad A, Ghasemzadeh Rahbardar M, Hosseinzadeh H (2023). Effect of trans-sodium crocetinate on contrast-induced cytotoxicity in HEK-293 cells. Iran J Basic Med Sci.

[55] Mohammadzadeh L, Ghasemzadeh Rahbardar M, Razavi BM, Hosseinzadeh H (2022). Crocin protects malathion-induced striatal biochemical deficits by inhibiting apoptosis and increasing α-synuclein in rats’ striatum. J Mol Neurosci.

[56] Rajabian F, Razavi BM, Mehri S, Amouian S, Ghasemzadeh Rahbardar M, Khajavi Rad A (2025). Evaluation of pathways involved in the protective effect of trans sodium crocetinate against contrast-induced nephropathy in rats. Naunyn Schmiedebergs Arch Pharmacol.

[57] Naraki GA, Fazeli Kakhki H, Qoorchi Moheb Seraj F, Naraki K, Ghasemzadeh Rahbardar M (2025). The effect of Crocus sativus L(saffron) and Rosmarinus officinalis L (rosemary) in hepatocellular carcinoma: A narrative review of current evidence and prospects. Iran J Basic Med Sci.

[58] Ghasemzadeh Rahbardar M, Hosseinzadeh H (2024). Therapeutic potential of hypnotic herbal medicines: A comprehensive review. Phytother Res.

[59] Hosseini SA, Zilaee M, Shoushtari MH, Ghasemi Dehcheshmeh M (2018). An evaluation of the effect of saffron supplementation on the antibody titer to heat-shock protein (HSP) 70, hsCRP and spirometry test in patients with mild and moderate persistent allergic asthma: A triple-blind, randomized placebo-controlled trial. Respir Med.

[60] Zilaee M, Hosseini SA, Jafarirad S, Abolnezhadian F, Cheraghian B, Namjoyan F (2019). An evaluation of the effects of saffron supplementation on the asthma clinical symptoms and asthma severity in patients with mild and moderate persistent allergic asthma: a double-blind, randomized placebo-controlled trial. Respir Res.

[61] Hosseini SA, Jamshidnezhad A, Zilaee M, Fouladi Dehaghi B, Mohammadi A, Hosseini SM (2020). Neural network-based clinical prediction system for identifying the clinical effects of saffron (Crocus sativus L) supplement therapy on allergic asthma: Model evaluation study. JMIR Med Inform.

[62] Ghobadi H, Abdollahi N, Madani H, Aslani MR (2022). Effect of crocin from saffron (Crocus sativus L) supplementation on oxidant/antioxidant markers, exercise capacity, and pulmonary function tests in COPD patients: A randomized, double-blind, placebo-controlled trial. Front Pharmacol.

[63] Aslani MR, Abdollahi N, Matin S, Zakeri A, Ghobadi H (2023). Effect of crocin of Crocus sativus L on serum inflammatory markers (IL-6 and TNF-α) in chronic obstructive pulmonary disease patients: a randomised, double-blind, placebo-controlled trial. Br J Nutr.

[64] Verma RK, Kumari P, Maurya RK, Kumar V, Verma R, Singh RK (2018). Medicinal properties of turmeric (Curcuma longa ): A review. Int J Chem Stud.

[65] Razavi BM, Ghasemzadeh Rahbardar M, Hosseinzadeh H (2021). A review of therapeutic potentials of turmeric (Curcuma longa) and its active constituent, curcumin, on inflammatory disorders, pain, and their related patents. Phytother Res.

[66] Ghasemzadeh Rahbardar M, Taghavizadeh Yazdi ME, Beigoli S, Amin H, Boskabady MH (2025). Exploring the role of curcumin, nanocurcumin, and a PPAR agonist in preventing paraquat-induced systemic inflammation and oxidative stress in rats. Iran J Basic Med Sci.

[67] Yang Q-Q, Cheng L-Z, Zhang T, Yaron S, Jiang H-X, Sui Z-Q (2020). Phenolic profiles, antioxidant, and antiproliferative activities of turmeric (Curcuma longa). Ind Crops Prod.

[68] Samanta S, Jyothi Y, Bihani M, Gurung S, Chettri R, Mahato AK (2024). Unveiling potent anti-asthmatic effect of curcumin in combination with salmeterol in Swiss Albino mice. Uttar Pradesh Journal of Zoology.

[69] Boskabady M, Khazdair M, Memarzia A, Behrouz S, Ghlamnezhad Z, Choudhary MI, Yousuf S (2021). Pharmacological effects of Curcuma longa, focused on anti-inflammatory, antioxidant and immunomodulatory effects. Science of Spices and Culinary Herbs-Latest Laboratory, Pre-clinical, and Clinical Studies.

[70] Memarzia A, Khazdair MR, Behrouz S, Gholamnezhad Z, Jafarnezhad M, Saadat S (2021). Experimental and clinical reports on anti-inflammatory, antioxidant, and immunomodulatory effects of Curcuma longa and curcumin, an updated and comprehensive review. BioFactors.

[71] Memarzia A, Saadat S, Behrouz S, Boskabady MH (2022). Curcuma longa and curcumin affect respiratory and allergic disorders, experimental and clinical evidence: A comprehensive and updated review. Biofactors.

[72] Boskabady MH, Shakeri F, Naghdi F The effects of Curcuma Longa L and its constituents in respiratory disorders and molecular mechanisms of their action. Studies in Natural Products Chemistry.

[73] Ghasemzadeh Rahbardar M, Hosseinzadeh H (2024). The ameliorative effect of turmeric (Curcuma longa Linn) extract and its major constituent, curcumin, and its analogs on ethanol toxicity. Phytother Res.

[74] Abidi A, Gupta S, Agarwal M, Bhalla HL, Saluja M (2014). Evaluation of efficacy of curcumin as an add-on therapy in patients of bronchial asthma. J Clin Diagn Res.

[75] Emami B, Shakeri F, Gholamnezhad Z, Saadat S, Boskabady M, Azmounfar V (2020). Calcium and potassium channels are involved in curcumin relaxant effect on tracheal smooth muscles. Pharm Biol.

[76] Khdair SA, Abdulridha MK, Fatah MA (2019). Effect of curcumin supplement on pulmonary functions, total and differential white blood cell count, serum level of leptin and body mass index in a sample of Iraqi patients with chronic bronchial asthma. Al-Mustansiriyah J Sci.

[77] Sura Abbas K, Manal Khalid A, Mostafa Abdalfatah S (2021). The effect of curcumin adjuvant therapy on pulmonary function and levels of interleukin-6 (IL-6) and superoxide dismutase-3 (EC-SOD3) in patients with chronic bronchial asthma. Indones J Pharm.

[78] Panahi Y, Ghanei M, Bashiri S, Hajihashemi A, Sahebkar A (2015). Short-term curcuminoid supplementation for chronic pulmonary complications due to sulfur mustard intoxication: Positive results of a randomized double-blind placebo-controlled trial. Drug Res.

[79] Nakisa N, Ghasemzadeh Rahbardar M (2021). Evaluating the probable effects of the COVID-19 epidemic detraining on athletes’ physiological traits and performance. Apunts Sports Med.

[80] Valizadeh H, Abdolmohammadi-Vahid S, Danshina S, Ziya Gencer M, Ammari A, Sadeghi A (2020). Nano-curcumin therapy, a promising method in modulating inflammatory cytokines in COVID-19 patients. Int Immunopharmacol.

[81] Ahmadi R, Salari S, Sharifi MD, Reihani H, Rostamiani MB, Behmadi M (2021). Oral nano-curcumin formulation efficacy in the management of mild to moderate outpatient COVID-19: A randomized triple-blind placebo-controlled clinical trial. Food Sci Nutr.

[82] Dabidi Roshan V, Noorbakhsh S, Ziatabar Ahmadi SF, Nikseresht M (2024). The physiological impacts of the low-and moderate-volume-HIIT with or without nano-curcumin supplement in women in menopause with obesity. Adv Public Health.

[83] Ng TP, Nyunt SZ, Gao Q, Gwee X, Chua DQL, Yap KB (2024). Curcumin-rich curry consumption and life expectancy: Singapore longitudinal ageing study. Geroscience.

[84] Sharma S (2021). A review on Eucalyptus globulus–an authentic herb. J Pharm Res Int.

[85] Balčiūnaitienė A, Liaudanskas M, Puzerytė V, Viškelis J, Janulis V, Viškelis P (2022). Eucalyptus globulus and Salvia officinalis extracts mediated green synthesis of silver nanoparticles and their application as an antioxidant and antimicrobial agent. Plants.

[86] Mworia JK, Kibiti CM, Ngeranwa JJ, Ngugi MP (2020). Analgesic potential of dichloromethane leaf extracts of Eucalyptus globulus (Labill) and Senna didymobotrya (Fresenius) in mice models. J Herbmed Pharmacol.

[87] Arooj B, Asghar S, Saleem M, Khalid SH, Asif M, Chohan T (2023). Anti-inflammatory mechanisms of eucalyptol rich Eucalyptus globulus essential oil alone and in combination with flurbiprofen. Inflammopharmacology.

[88] Amini N, Yazdannik A, Safarabadi M, Harorani M, Rezaei K (2022). Effect of nebulized Eucalyptus on arterial blood gases and physiologic indexes of mechanical ventilated patients: a randomized clinical trial. J Caring Sci.

[89] Öztürk GY, Safçi SB (2022). Effects of eucalyptus essential oil in post-COVID syndrome: A pilot study. J Immunol Clin Microbiol.

[90] Amini N, Yazdannik A, Safarabadi M, Harorani M, Rezaei K (2022). Effect of Nebulized Eucalyptus on Arterial Blood Gases and Physiologic Indexes of Mechanical Ventilated Patients: A Randomized Clinical Trial. J Caring Sci.

[91] Worth H, Schacher C, Dethlefsen U (2009). Concomitant therapy with Cineole (Eucalyptole) reduces exacerbations in COPD: A placebo-controlled double-blind trial. Respir Res.

[92] Worth H, Dethlefsen U (2012). Patients with asthma benefit from concomitant therapy with cineole: a placebo-controlled, double-blind trial. J Asthma.

[93] Hudz N, Kobylinska L, Pokajewicz K, Horčinová Sedláčková V, Fedin R, Voloshyn M (2023). Mentha piperita: Essential oil and extracts, their biological activities, and perspectives on the development of new medicinal and cosmetic products. Molecules.

[94] Park N, Chung JY, Kim MH, Yang WM (2022). Protective effects of inhalation of essential oils from Mentha piperita leaf on tight junctions and inflammation in allergic rhinitis. Front Allergy.

[95] Desam NR, Al-Rajab AJ, Sharma M, Mylabathula MM, Gowkanapalli RR, Albratty M (2019). Chemical constituents, in vitro antibacterial and antifungal activity of Mentha× Piperita L (peppermint) essential oils. J King Saud Univ Sci.

[96] Saravanan R, Natesan R, Samiappan SC, Ramalingam S (2021). Anti-oxidant, anti-bacterial and anti-cancer activity of Mentha piperita against MCF-7 cells. Biomed Pharmacol J.

[97] Kehili S, Boukhatem MN, Belkadi A, Ferhat MA, Setzer WN (2020). Peppermint (Mentha piperita L ) essential oil as a potent anti-inflammatory, wound healing and anti-nociceptive drug. Eur J Biol Res.

[98] Silva WMF, Bona NP, Pedra NS, Cunha KFD, Fiorentini AM, Stefanello FM (2022). Risk assessment of in vitro cytotoxicity, antioxidant and antimicrobial activities of Mentha piperita L essential oil. J Toxicol Environ Health.

[99] Kim MH, Park SJ, Yang WM (2020). Inhalation of essential oil from Mentha piperita ameliorates PM10-exposed asthma by targeting IL-6/JAK2/STAT3 pathway based on a network pharmacological analysis. Pharmaceuticals.

[100] Meamarbashi A, Rajabi A (2013). The effects of peppermint on exercise performance. J Int Soc Sports Nutr.

[101] Shepherd K, Peart DJ (2017). Aerobic capacity is not improved following 10-day supplementation with peppermint essential oil. Appl Physiol Nutr Metab.

[102] Nishino T, Tagaito Y, Sakurai Y (1997). Nasal inhalation of l-menthol reduces respiratory discomfort associated with loaded breathing. Am J Respir Crit Care Med.

[103] Haidl P, Kemper P, Butnarasu SJ, Klauke M, Wehde H, Köhler D (2001). Does the inhalation of a 1% L-menthol solution in the premedication of fiberoptic bronchoscopy affect coughing and the sensation of dyspnea?. Pneumologie.

[104] Kanezaki M, Terada K, Ebihara S (2020). Effect of olfactory stimulation by L-menthol on laboratory-induced dyspnea in COPD. Chest.

[105] Sato N, Ogura R, Iwanami Y, Okuni I, Ebihara S (2023). L-menthol olfactory stimulation reduced dyspnea sensation during the 6 min walk test in patients with chronic breathlessness syndrome: A pilot study. J Clin Med.

[106] Tsutsumi Y, Momma H, Ebihara S, Nagatomi R (2023). L-menthol administration facilitates breathing comfort during exhaustive endurance running and improves running capacity in well-trained runners: A randomized crossover study. Eur J Sport Sci.

[107] Vafaeipour Z, Ghasemzadeh Rahbardar M, Hosseinzadeh H (2023). Effect of saffron, black seed, and their main constituents on inflammatory cytokine response (mainly TNF-α) and oxidative stress status: an aspect on pharmacological insights. Naunyn Schmiedebergs Arch Pharmacol.

[108] Hosseini A, Mehri S, Aminifard T, Ghasemzadeh Rahbardar M, Nouripor S, Khajavi Rad A (2024). Renoprotective effect of thymoquinone against rhabdomyolysis-induced acute kidney injury in the rat model. Iran J Basic Med Sci.

[109] Fadishei M, Ghasemzadeh Rahbardar M, Imenshahidi M, Mohajeri A, Razavi BM, Hosseinzadeh H (2021). Effects of Nigella sativa oil and thymoquinone against bisphenol A-induced metabolic disorder in rats. Phytother Res.

[110] Khazdair MR, Amirabadizadeh A (2022). Therapeutic effects of Nigella sativa on asthma: a systematic review of clinical trial. Physiol Pharmacol.

[111] Gholamnezhad Z, Keyhanmanesh R, Boskabady MH (2015). Anti-inflammatory, antioxidant, and immunomodulatory aspects of Nigella sativa for its preventive and bronchodilatory effects on obstructive respiratory diseases: A review of basic and clinical evidence. J Funct Foods.

[112] Saadat S, Aslani MR, Ghorani V, Keyhanmanesh R, Boskabady MH (2021). The effects of Nigella sativa on respiratory, allergic and immunologic disorders, evidence from experimental and clinical studies, a comprehensive and updated review. Phytother Res.

[113] Bouti K, Rhorfi IA, Mzouri M, Abid A, Alaoui Tahiri K (2013). Exogenous lipoid pneumonia caused by Nigella sativa oil – A case report. Egypt J Chest Dis Tuberc.

[114] Boskabady MH, Javan H, Sajady M, Rakhshandeh H (2007). The possible prophylactic effect of Nigella sativa seed extract in asthmatic patients. Fundam Clin Pharmacol.

[115] Boskabady MH, Mohsenpoor N, Takaloo L (2010). Antiasthmatic effect of Nigella sativa in airways of asthmatic patients. Phytomedicine.

[116] Al-Jawad FH, Al-Razzuqi RA, Hashim H, Ismael AH (2012). Broncho-relaxant activity of Nigella sativa versus anthemis nobilis in chronic bronchial asthma; a comparative study of efficacy. IOSR J Pharm.

[117] Koshak A, Wei L, Koshak E, Wali S, Alamoudi O, Demerdash A (2017). Nigella sativa supplementation improves asthma control and biomarkers: A randomized, double-blind, placebo-controlled trial. Phytother Res.

[118] Salem AM, Bamosa AO, Qutub HO, Gupta RK, Badar A, Elnour A (2017). Effect of Nigella sativa supplementation on lung function and inflammatory mediatorsin partly controlled asthma: a randomized controlled trial. Ann Saudi Med.

[119] Boskabady MH, Farhadi J (2008). The possible prophylactic effect of Nigella sativa seed aqueous extract on respiratory symptoms and pulmonary function tests on chemical war victims: a randomized, double-blind, placebo-controlled trial. J Altern Complement Med.

[120] Al-Azzawi MA, AboZaid MMN, Ibrahem RAL, Sakr MA (2020). Therapeutic effects of black seed oil supplementation on chronic obstructive pulmonary disease patients: A randomized controlled double blind clinical trial. Heliyon.

[121] Said SA, Abdulbaset A, El-Kholy AA, Besckales O, Sabri NA (2022). The effect of Nigella sativa and vitamin D3 supplementation on the clinical outcome in COVID-19 patients: A randomized controlled clinical trial. Front Pharmacol.

[122] Ghasemzadeh Rahbardar M, Hosseinzadeh H (2024). Toxicity and safety of rosemary (Rosmarinus officinalis): a comprehensive review. Naunyn Schmiedebergs Arch Pharmacol.

[123] Ghasemzadeh MR, Amin B, Mehri S, Mirnajafi-Zadeh SJ, Hosseinzadeh H (2016). Effect of alcoholic extract of aerial parts of Rosmarinus officinalis on pain, inflammation and apoptosis induced by chronic constriction injury (CCI) model of neuropathic pain in rats. J Ethnopharmacol.

[124] Nakisa N, Ghasemzadeh Rahbardar M (2022). Therapeutic potential of rosemary (Rosmarinus officinalis L ) on sports injuries: A review of patents. Res J Pharmacogn.

[125] Ghasemzadeh Rahbardar M, Amin B, Mehri S, Mirnajafi-Zadeh SJ, Hosseinzadeh H (2017). Anti-inflammatory effects of ethanolic extract of Rosmarinus officinalis and rosmarinic acid in a rat model of neuropathic pain. Biomed Pharmacother.

[126] Ghasemzadeh Rahbardar M, Hemadeh B, Razavi BM, Eisvand F, Hosseinzadeh H (2022). Effect of carnosic acid on acrylamide induced neurotoxicity: in vivo and in vitro experiments. Drug Chem Toxicol.

[127] Alavi MS, Fanoudi S, Ghasemzadeh Rahbardar M, Mehri S, Hosseinzadeh H (2021). An updated review of protective effects of rosemary and its active constituents against natural and chemical toxicities. Phytother Res.

[128] Ghasemzadeh Rahbardar M, Ferns GA, Ghayour Mobarhan M (2025). Assessing the efficacy of herbal supplements for managing obesity: A comprehensive review of global clinical trials. Iran J Basic Med Sci.

[129] Ghasemzadeh Rahbardar M, Eisvand F, Rameshrad M, Razavi BM, Tabatabaee Yazdi A, Hosseinzadeh H (2024). Carnosic acid mitigates doxorubicin-induced cardiac toxicity: Evidence from animal and cell model investigations. Iran J Basic Med Sci.

[130] Rahbardar MG, Eisvand F, Rameshrad M, Razavi BM, Hosseinzadeh H (2022). In vivo and in vitro protective effects of rosmarinic acid against doxorubicin-induced cardiotoxicity. Nutr Cancer.

[131] Nakisa N, Rahbardar MG (2021). Action mechanisms of antirheumatic herbal medicines. Rheumatoid arthritis.

[132] Rahbardar MG, Amin B, Mehri S, Mirnajafi-Zadeh SJ, Hosseinzadeh H (2018). Rosmarinic acid attenuates development and existing pain in a rat model of neuropathic pain: An evidence of anti-oxidative and anti-inflammatory effects. Phytomedicine.

[133] Rahbardar MG, Hosseinzadeh H, Martin CR, Hunter L-A, Patel VB, Preedy VR, Rajendram R (2021). Chapter 47 - Mechanisms of action of herbal antidepressants. The Neuroscience of Depression.

[134] Farhadi F, Baradaran Rahimi V, Mohamadi N, Askari VR (2023). Effects of rosmarinic acid, carnosic acid, rosmanol, carnosol, and ursolic acid on the pathogenesis of respiratory diseases. Biofactors.

[135] Ghasemzadeh Rahbardar M, Hosseinzadeh H (2020). Effects of rosmarinic acid on nervous system disorders: an updated review. Naunyn Schmiedebergs Arch Pharmacol.

[136] Ghasemzadeh Rahbardar M, Hosseinzadeh H (2020). Therapeutic effects of rosemary (Rosmarinus officinalis L ) and its active constituents on nervous system disorders. Iran J Basic Med Sci.

[137] Mirsadraee M, Tavakoli A, Ghorani V, Ghaffari S (2018). Effects of Rosmarinus officinalis and Platanus orientalis extracts on asthmatic subjects resistant to routine treatments. Avicenna J Phytomed.

[138] Momeni Safarabadi A, Gholami M, Kordestani-Moghadam P, Ghaderi R, Birjandi M (2024). The effect of rosemary hydroalcoholic extract on cognitive function and activities of daily living of patients with chronic obstructive pulmonary disease (COPD): A clinical trial. Explore (NY).

[139] Juergens UR, Dethlefsen U, Steinkamp G, Gillissen A, Repges R, Vetter H (2003). Anti-inflammatory activity of 1 8-cineol (eucalyptol) in bronchial asthma: a double-blind placebo-controlled trial. Respir Med.

[140] Patil SM, Ramu R, Shirahatti PS, Shivamallu C, Amachawadi RG (2021). A systematic review on ethnopharmacology, phytochemistry and pharmacological aspects of Thymus vulgaris Linn. Heliyon.

[141] Pandiyan I, Sri SD, Indiran MA, Rathinavelu PK, Prabakar J, Rajeshkumar S (2022). Antioxidant, anti-inflammatory activity of Thymus vulgaris-mediated selenium nanoparticles: An in vitro study. J Conserv Dent.

[142] Khazdair MR, Gholamnezhad Z, Rezaee R, Boskabady MH (2021). Immuno-modulatory and anti-inflammatory effects of Thymus vulgaris, Zataria multiflora, and Portulaca oleracea and their constituents. Pharmacol Res - Mod Chin Med.

[143] Ghahremani-Chabok A, Bagheri-Nesami M, Shorofi SA, Mousavinasab SN, Gholipour-Baradari A, Saeedi M (2021). The effects of Thymus vulgaris inhalation therapy on airway status and oxygen saturation of patients under mechanical ventilation: A randomized clinical trial. Adv Integr Med.

[144] Wirtu SF, Ramaswamy K, Maitra R, Chopra S, Mishra AK, Jule LT (2024). Isolation, characterization and antimicrobial activity study of Thymus vulgaris. Sci Rep.

[145] Afonso AF, Pereira OR, Cardoso SM (2020). Health-promoting effects of Thymus phenolic-rich extracts: Antioxidant, anti-inflammatory and antitumoral properties. Antioxidants.

[146] Eskandarpour E, Ahadi A, Jazani AM, Azgomi RND, Molatefi R (2024). Thymus vulgaris ameliorates cough in children with asthma exacerbation: a randomized, triple-blind, placebo-controlled clinical trial. Allergol Immunopathol.

[147] Öner U, Cengiz Z (2024). The effects of aromatherapy with thyme oil on disease symptoms, vital findings, and hemodynamic parameters in COVID-19 patients. Explore (NY).

[148] Sajed H, Sahebkar A, Iranshahi M (2013). Zataria multiflora Boiss. (Shirazi thyme)—An ancient condiment with modern pharmaceutical uses. J Ethnopharmacol.

[149] Arab Z, Hosseini M, Marefati N, Beheshti F, Anaeigoudari A, Sadeghnia HR (2022). Neuroprotective and memory enhancing effects of Zataria multiflora in lipopolysaccharide-treated rats. Vet Res Forum.

[150] Ghasemzadeh Rahbardar M, Khosravi R, Beigoli S, Sarbaz P, Amirahmadi S, Hosseini M (2025). Examining the preventive effect of Zataria multiflora Boiss on paraquat-induced behavioral impairment and hippocampal oxidative stress. Toxicol Environ Health Sci.

[151] Golkar P, Mosavat N, Jalali SAH (2020). Essential oils, chemical constituents, antioxidant, antibacterial and in vitro cytotoxic activity of different Thymus species and Zataria multiflora collected from Iran. S Afr J Bot.

[152] Hashemi SA, Azadeh S, Nouri BM, Navai RA (2017). Review of pharmacological effects of Zataria multiflora Boiss (thyme of Shiraz). International Journal of Medical Research & Health Sciences.

[153] Boskabady M, Alavinezhad A, Boskabady MH (2021). Zataria multiflora induced bronchodilatoion comparable to theophylline syrup in asthmatic patients. Explore.

[154] Hosseini M, Arab Z, Beheshti F, Anaeigoudari A, Shakeri F, Rajabian A (2023). Zataria multiflora and its constituent, carvacrol, counteract sepsis-induced aortic and cardiac toxicity in rat: Involvement of nitric oxide and oxidative stress. Anim Models Exp Med.

[155] Alavinezhad A, Hedayati M, Boskabady MH (2017). The effect of Zataria multiflora and carvacrol on wheezing, FEV1 and plasma levels of nitrite in asthmatic patients. Avicenna J Phytomed.

[156] Alavinezhad A, Ghorani V, Rajabi O, Boskabady MH (2020). Zataria multiflora affects clinical symptoms, oxidative stress and cytokines in asthmatic patient: A randomized, double blind, placebo-controlled, phase II clinical trial. Cytokine.

[157] Boskabady MH, Tabanfar H (2011). Effect of Zataria multiflora Bois on histamine (H1) receptor of guinea pig tracheal chains. Indian J Exp Biol.

[158] Jafari Z, Boskabady MH, Pouraboli I, Babazade B (2011). Zataria multiflora Boiss inhibits muscarinic receptors of incubated tracheal smooth muscle with propranolol. Avicenna J Phytomed.

[159] Boskabady MH, Tabanfar H, Gholamnezhad Z, Sadeghnia HR (2012). Inhibitory effect of Zataria multiflora Boiss and carvacrol on histamine (H(1) ) receptors of guinea-pig tracheal chains. Fundam Clin Pharmacol.

[160] Khazdair MR, Rajabi O, Balali-Mood M, Beheshti F, Boskabady MH (2018). The effect of Zataria multiflora on pulmonary function tests, hematological and oxidant/antioxidant parameters in sulfur mustard exposed veterans, a randomized doubled-blind clinical trial. Environ Toxicol Pharmacol.

[161] Khazdair MR, Rezaeetalab F, Rafatpanah H, Boskabady MH (2020). The effect of Zataria multiflora on inflammatory cytokine and respiratory symptoms in veterans exposed to sulfur mustard. Environ Sci Pollut Res Int.

[162] Khazdair MR, Ghorani V, Alavinezhad A, Boskabady MH (2020). Effect of Zataria multiflora on serum cytokine levels and pulmonary function tests in sulfur mustard-induced lung disorders: A randomized double-blind clinical trial. J Ethnopharmacol.

[163] Ghorani V, Rajabi O, Mirsadraee M, Rezaeitalab F, Saadat S, Boskabady MH (2020). A randomized, doubled-blind clinical trial on the effect of Zataria multiflora on clinical symptoms, oxidative stress, and C-reactive protein in COPD patients. J Clin Pharmacol.

[164] Boskabady MH, Mehrjardi SS, Rezaee A, Rafatpanah H, Jalali S (2013). The impact of Zataria multiflora Boiss extract on in vitro and in vivo Th1/Th2 cytokine (IFN-γ/IL4) balance. J Ethnopharmacol.

[165] Boskabady MH, Tabatabaee A, Jalali S (2014). Potential effect of the extract of Zataria multiflora and its constituent, carvacrol, on lung pathology, total and differential WBC, IgE and eosinophil peroxidase levels in sensitized guinea pigs. J Funct Foods.

[166] Boskabady MH, Jalali S, Farkhondeh T, Byrami G (2014). The extract of Zataria multiflora affect tracheal responsiveness, serum levels of NO, nitrite, PLA2, TP and histamine in sensitized Guinea pigs. J Ethnopharmacol.

[167] Kianmehr M, Rezaei A, Boskabady MH (2016). Effect of carvacrol on various cytokines genes expression in splenocytes of asthmatic mice. Iran J Basic Med Sci.

[168] Kianmehr M, Rezaei A, Hosseini M, Khazdair MR, Rezaee R, Askari VR (2017). Immunomodulatory effect of characterized extract of Zataria multiflora on Th1, Th2 and Th17 in normal and Th2 polarization state. Food ChemToxicol.

[169] Ghorani V, Khazdair MR, Mirsadraee M, Rajabi O, Boskabady MH (2022). The effect of two-month treatment with Zataria multiflora on inflammatory cytokines, pulmonary function testes and respiratory symptoms in patients with chronic obstructive pulmonary disease (COPD). J Ethnopharmacol.

[170] Boskabady MH, Gholami Mhtaj L (2014). Effect of the Zataria multiflora on systemic inflammation of experimental animals model of COPD. Biomed Res Int.

[171] Gholami Mahtaj L, Boskabady MH, Mohamadian Roshan N (2015). The effect of Zataria multiflora and its constituent, carvacrol, on tracheal responsiveness and lung pathology in Guinea pig model of COPD. Phytother Res.

[172] Boskabady MH, Jalali S (2013). Effect of carvacrol on tracheal responsiveness, inflammatory mediators, total and differential WBC count in blood of sensitized guinea pigs. Exp Biol Med.

[173] Jalali S, Boskabady MH, Rohani AH, Eidi A (2013). The effect of carvacrol on serum cytokines and endothelin levels of ovalbumin sensitized guinea-pigs. Iran J Basic Med Sci.

[174] Boskabady MH, Jalali S, Yahyazadeh N, Boskabady M (2016). Carvacrol attenuates serum levels of total protein, phospholipase A2 and histamine in asthmatic guinea pig. Avicenna J Phytomed.

[175] Khazdair MR, Alavinezhad A, Boskabady MH (2018). Carvacrol ameliorates haematological parameters, oxidant/antioxidant biomarkers and pulmonary function tests in patients with sulphur mustard-induced lung disorders: A randomized double-blind clinical trial. J Clin Pharm Ther.

[176] Khazdair MR, Boskabady MH (2019). A double-blind, randomized, placebo-controlled clinical trial on the effect of carvacrol on serum cytokine levels and pulmonary function tests in sulfur mustard induced lung injury. Cytokine.

[177] Khazdair MR, Boskabady MH (2019). The effect of carvacrol on inflammatory mediators and respiratory symptoms in veterans exposed to sulfur mustard, a randomized, placebo-controlled trial. Respir Med.

[178] Ghasemzadeh Rahbardar M, Razavi BM, Naraki K, Hosseinzadeh H (2023). Therapeutic effects of minocycline on oleic acid-induced acute respiratory distress syndrome (ARDS) in rats. Naunyn Schmiedebergs Arch Pharmacol.

[179] Bezerra FS, Lanzetti M, Nesi RT, Nagato AC, Silva CPE, Kennedy-Feitosa E (2023). Oxidative Stress and Inflammation in Acute and Chronic Lung Injuries. Antioxidants (Basel).

[180] Aghasafari P, George U, Pidaparti R (2019). A review of inflammatory mechanism in airway diseases. Inflamm Res.

[181] Verma G, Dhawan M, Saied AA, Kaur G, Kumar R, Emran TB (2023). Immunomodulatory approaches in managing lung inflammation in COVID-19: A double-edge sword. Immun Inflamm Dis.

[182] Millar JE, Craven TH, Shankar-Hari M (2024). Steroids and Immunomodulatory Therapies for Acute Respiratory Distress Syndrome. Clin Chest Med.

[183] Ghorani V, Alavinezhad A, Rajabi O, Mohammadpour AH, Boskabady MH (2021). Safety and tolerability of carvacrol in healthy subjects: a phase I clinical study. Drug Chem Toxicol.

[184] Ghorani V, Boskabady M, Boskabady MH (2019). Effect of carvacrol on pulmonary function tests, and total and differential white blood cell counts in healthy volunteers: A randomized clinical trial. Avicenna J Phytomed.

